# Histamine stimulates human microglia to alter cellular prion protein expression via the HRH2 histamine receptor

**DOI:** 10.1038/s41598-024-75982-1

**Published:** 2024-10-26

**Authors:** Marcus Pehar, Melissa Hewitt, Ashley Wagner, Jagdeep K. Sandhu, Aria Khalili, Xinyu Wang, Jae-Young Cho, Valerie L. Sim, Marianna Kulka

**Affiliations:** 1 Quantum and Nanotechnologies Research Centre, National Research Council Canada, Edmonton, AB Canada; 2 Neuroscience and Mental Health Institute, Faculty of Medicine and Dentistry, University of Alberta, Edmonton, AB Canada; 3 Centre for Prions and Protein Folding Diseases, University of Alberta, Edmonton, AB Canada; 4 Human Health Therapeutics Research Centre, National Research Council Canada, Ottawa, ON Canada; 5 Department of Mechanical Engineering, University of Alberta, Edmonton, AB Canada; 6 Department of Medicine, University of Alberta, Edmonton, AB Canada; 7 Department of Medical Microbiology and Immunology, University of Alberta, Edmonton, AB Canada

**Keywords:** Prion protein, Microglia, Histamine, Mast cell, Degranulation, Neuroinflammation, Cell biology, Neuroscience, Biomarkers

## Abstract

Although the cellular prion protein (PrP^C^) has been evolutionarily conserved, the role of this protein remains elusive. Recent evidence indicates that PrP^C^ may be involved in neuroinflammation and the immune response in the brain, and its expression may be modified via various mechanisms. Histamine is a proinflammatory mediator and neurotransmitter that stimulates numerous cells via interactions with histamine receptors 1-4 (HRH1-4). Since microglia are the innate immune cells of the central nervous system, we hypothesized that histamine-induced stimulation regulates the expression of PrP^C^ in human-derived microglia. The human microglial clone 3 (HMC3) cell line was treated with histamine, and intracellular calcium levels were measured via a calcium flux assay. Cytokine production was monitored by enzyme-linked immunosorbent assay (ELISA). Western blotting and quantitative reverse transcription-polymerase chain reaction were used to determine protein and gene expression of HRH1-4. Flow cytometry and western blotting were used to measure PrP^C^ expression levels. Fluorescence microscopy was used to examine Iba-1 and PrP^C^ localization. HMC3 cells stimulated by histamine exhibited increased intracellular calcium levels and increased release of IL-6 and IL-8, while also modifying PrP^C^ localization. HMC3 stimulated with histamine for 6 and 24 hours exhibited increased surface PrP^C^ expression. Specifically, we found that stimulation of the HRH2 receptor was responsible for changes in surface PrP^C^. Histamine-induced increases in surface PrP^C^ were attenuated following inhibition of the HRH2 receptor via the HRH2 antagonist ranitidine. These changes were unique to HRH2 activation, as stimulation of HRH1, HRH3, or HRH4 did not alter surface PrP^C^. Prolonged stimulation of HMC3 decreased PrP^C^ expression following 48 and 72 hours of histamine stimulation. HMC3 cells can be stimulated by histamine to undergo intracellular calcium influx. Surface expression levels of PrP^C^ on HMC3 cells are altered by histamine exposure, primarily mediated by HRH2. While histamine exposure also increases release of IL-6 and IL-8 in these cells, this cytokine release is not fully dependent on PrP^C^ levels, as IL-6 release is only partially reduced and IL-8 release is unchanged under the conditions of HRH2 blockade that prevent PrP^C^ changes. Overall, this suggests that PrP^C^ may play a role in modulating microglial responses.

**Supplementary Information:**

The online version contains supplementary material available at 10.1038/s41598-024-75982-1.

## Introduction

Microglia are ubiquitously found throughout the brain and are considered the resident innate immune cells of the central nervous system (CNS). In healthy adult brains, these cells dynamically monitor their local milieu, mediating homeostasis and regulating immune function^1^. Microglia enrich and support healthy brain function and CNS regulation via the release of cytokines, chemokines, and various trophic factors in response to damage- and pathogen-associated molecular patterns presented by harmful stimuli and debris^2^. These proinflammatory mediators can then accumulate in specific tissue regions, inducing an inflammatory cascade and stimulating more microglia to participate in inflammation and immune regulation.

Histamine is a neurotransmitter and neuromodulator in the CNS that participates in numerous brain functions. This mediator is involved in neuroinflammation and contributes to both innate and adaptive immune responses^3^. The sources of histamine in the body and CNS include, but are not limited to mast cells, which release histamine during degranulation^4^, and the tuberomammillary nucleus, which consists of various histaminergic neurons responsible for immune function, cognition, wakefulness, and neurotransmission^5^. Histamine acts via interaction with four G protein-coupled histamine receptors (GPCRs): HRH1, HRH2, HRH3, and HRH4. The HRH1 and HRH2 receptors are primarily involved in proinflammatory actions^6,7^, whereas physiological actions such as cytokine release and neuroinflammation regulation are mediated by HRH2 and HRH3^3,6^. The HRH4 receptor was the last to be discovered and, although the function of this receptor is not yet fully understood, HRH4 is thought to be involved in mediating mast cell chemotaxis^8^ and stimulating the immune response in asthma, potentially through its neuroinflammatory capacity^9^.

Recently, studies on microglia have shown that histamine alters microglial reactivity and stimulates these cells in neurological disease^10^. Microglia can be exposed to histamine via various mechanisms, as this molecule is frequently found in the CNS as a neurotransmitter. Recent studies have shown that mast cells can interact with microglia via the release of histamine^11^, potentially resulting in changes in microglial phenotypes^12^.

Studies have indicated that murine microglia react to histamine by increasing the release of the inflammatory mediators IL-1β^13^, TNF^3^, IL-6^14^, and various others^15^. Bader et al. were the first to demonstrate that histamine activates microglia^16^; this work used calcium imaging to highlight that primary rat microglia were reactive to histamine and increased their intracellular calcium concentrations after exposure. There is limited and contradictory evidence pertaining to the expression of histamine receptors on microglia; for example, functional HRH1 has been demonstrated in murine N9 microglia^15^ but not in primary mouse microglia^17^. Notwithstanding, microglial reactivity to histamine has been reported via all four histamine receptors in some capacity^15,17,18^.

The cellular prion protein (PrP^C^) is a small cell-surface glycoprotein ubiquitously expressed in the CNS^19,20^ and immune system^21–23^. A range of physiological functions have been reported for PrP^C^ and this protein functions as an important signalling coreceptor in the brain that may form signalling complexes with other receptors, such as gangliosides or microdomains common in immune cell activation^22^. Genomic and structural analysis of PrP^C^ has indicated that this protein is highly conserved among vertebrates^24^, suggesting that this protein has an evolutionary advantage. PrP^C^ is expressed at various levels in immune cells, and accordingly, there have been numerous proposed physiological roles for it, including mediating inflammation^25^ and immune cell activation^26^. The gene that encodes PrP^C^, *PRNP*, is expressed in neurons and glial cells of the CNS^27^, and PrP^C^ is thought to mediate inflammation and protection against pathological stresses such as oxidative damage^28^. PrP^C^ is expressed in mouse microglia, where it may play a role in the maintenance of these cells in their quiescent state and regulate microglial reactivity by modifying cellular homeostasis and promoting inflammation^20^. However, there is currently little understanding of PrP^C^ in human microglia and immune cells; the physiological role of this protein in human glial cells remains elusive.

In previous studies, stimulating BV-2 microglia with neurotoxins resulted in changes in *Prnp* gene expression^29^ and PrP^C^ protein expression^30^; PrP^C^ expression may be necessary for microglial stimulation and inflammation induction^31^. As histamine is a key regulator of neuroinflammation, we wanted to test the effects of histamine on microglial reactivity and investigate potential changes in PrP^C^ expression. In the present study, we hypothesized that human-derived microglia could be stimulated by the neurotransmitter and immune mediator histamine to alter the expression of PrP^C^, potentially revealing important new insights into PrP^C^ functions in the brain and neuroinflammation.

## Materials and methods

### Cell culture

HMC3 cells (ATCC, Manassas, VA, USA) were cultured in complete culture media consisting of minimum essential medium (MEM) (#11095080, Thermo Scientific, Waltham, MA, USA) supplemented with 10% heat-inactivated FBS (HyClone, Logan, UT, USA), 100 U/mL penicillin (Thermo Scientific), and 100 µg/mL streptomycin (Gibco, Waltham, MA, USA). Cells were maintained in a humidified atmosphere at 37^o^C and 5% CO_2_. HMC3 cells were cultured at 2 × 10^4^ cells/cm^2^ and, once reaching 80% confluency, were detached with a 5-minute treatment of 0.25% trypsin EDTA (Gibco) before the addition of complete culture media to deactivate the enzyme. Notably, microglia are known to react to serum^32^ and we have demonstrated that microglia in high-serum conditions secrete higher amounts of IL-8 and IL-6 cytokines (Supplementary 1) which may indicate a stimulated state. Accordingly, 24 hours prior to experiments, the complete culture media was replaced with MEM supplemented with 1% heat-inactivated FBS to limit the basal proliferation and cytokine secretion of cells and maintain a less stimulated microglial state.

The Laboratory of Allergic Disease 2 (LAD2) mast cell line, which was donated by Dr. Arnold Kischenbaum and Dr. Dean Metcalfe from the National Institute of Allergy and Infectious Disease, was cultured according to previously described protocols^23^. Brain tissue homogenates were obtained from the National Prion Disease Pathology Surveillance Centre (NPDPSC) at Case Western Reserve University (CWRU) in Cleveland, OH and prepared as previously described^33^.

## Treatments

Stock concentrations of histamine (0.1–1000 µM, Sigma, St. Louis, MO, USA) and LPS (1 µg/mL, Sigma) were prepared in sterile phosphate-buffered saline (PBS, Gibco) at pH 7.4 without calcium or magnesium and stored at -20^o^C. Histamine receptor agonists (HRH1: HTMT, HRH2: amthamine, HRH3: R-(-)-α-methylhistamine, and HRH4: 4-methylhistamine, Cayman Chemical Company, Ann Arbor, MI, USA) and antagonists (HRH1: clemastine, HRH2: ranitidine, HRH3: JNJ-5207852, and HRH4: JNJ-7777120, Cayman Chemical Company) were prepared in sterile phosphate-buffered saline (PBS, Gibco) at pH 7.4 without calcium or magnesium. For all experiments that included histamine receptor antagonists, HMC3 cells were seeded at relevant densities for 2 hours prior to antagonist treatment, and then left for an additional hour prior to histamine treatment.

## Cryo-scanning electron microscopy (Cryo-SEM)

HMC3 cells were cultured on a poly-D-lysine treated coverslip for 72 hours. The coverslip was then soaked in PBS buffer and gently blotted with filter paper to remove excess solution. The coverslip was rapidly plunged into liquid nitrogen and allowed to stabilize. Sublimation at -90°C, followed by platinum sputter-coating for 120 seconds, was performed using a Leica ACE 600. The sample was then transferred using the VCT100 (Leica) to a ZEISS NVision 40 for imaging at an accelerating voltage of 2 kV and a temperature of -140°C, utilizing a secondary electron (SE) detector.

## Quantitative reverse transcription-polymerase chain reaction (qRT‒PCR)

HMC3 cells were pelleted, and RNA was isolated using a combined TRIzol (Invitrogen) and column method as follows. First, the pellets were resuspended in TRIzol at 1.5 × 10^6^ cells/mL before the addition of chloroform (the ratio of TRIzol to chloroform was 1:0.167). The mixture was then spun at 12,000 × g for 5 min at 4 °C, after which the RNA-containing layer was removed. This layer was then added to an RNeasy Mini Kit (Qiagen) and an equal volume of 70% ethanol was added. The RNA and ethanol were vigorously mixed and washed according to the RNeasy^®^ protocol before RNA was eluted and RNA quality was assessed via NanoDrop One UV-Vis spectrophotometer (Thermo Scientific).

RNA (1 µg) was reverse transcribed with M-MLV reverse transcriptase (Invitrogen) according to manufacturer protocol (Invitrogen). Thirty nanograms of RNA per reaction was analysed by qRT‒PCR using PrimeTime Gene Expression Master Mix (IDT, Newark, NJ, USA) on a StepOnePlus real-time PCR instrument (Applied Biosystems). The primers used were human HRH1 (Hs.PT.58.39265294, IDT), HRH2 (Hs.PT.58.27972838, IDT), HRH3 (Hs.PT.58.38998576, IDT), and HRH4 (Hs.PT.58.4403324, IDT). GAPDH PrimeTime Probes (Mm.PT.58.30458786 and Mm.PT.39a.1, IDT) were used according to manufacturer protocol (IDT). The results are presented as the ΔCT relative to the GAPDH level.

## Metabolic analysis using XTT

HMC3 cells were seeded at a density of 1.0 × 10^4^ cells/mL in a 96-well plate and incubated in a humidified atmosphere of 5% CO_2_ at 37^o^C for 2 hours to adhere to the plate. The cells were then treated with histamine (0.1 µM-1000 µM) or LPS (0.1 µg/mL-50 µg/mL) and incubated for 24 hours at 37^o^C and 5% CO_2_. Metabolic activity was assessed via a Cell Proliferation Kit II (XTT Assay, Roche, Basel, Switzerland) according to the manufacturer’s instructions, and the results are presented as the percent metabolic activity relative to that of untreated cells.

### Cytokine enzyme-linked immunosorbent assays (ELISAs) and electrochemiluminescence

HMC3 cells were seeded at a density of 5.0 × 10^4^ cells/cm^2^ in a 6-well plate and incubated in a humidified atmosphere of 5% CO_2_ at 37^o^C for 2 hours to adhere to the plate. The cells were then treated with histamine (10 µM-1000 µM) and incubated for 24, 48, or 72 hours at 37^o^C and 5% CO_2_. For experiments involving antagonists, cells were treated with 100 µM of clemastine, ranitidine, JNJ-5207852, or JNJ-7777120 and incubated for 1 hour before treatment with 100 µM histamine. Cell-free supernatants were isolated and stored at -20^o^C. ELISA analysis for human IL-8 and IL-6 was performed using commercial ELISA kits (R&D Systems, Minneapolis, MIN, USA) and the plates were read using a VarioSkan Lux plate reader (Thermo Scientific). Additional cell-free supernatant samples were analysed via electrochemiluminescence as previously described^34^ to measure for the production of IL-8, IL-6, IL-10, IL-4, IFN-γ, and IL-12p70.

## Intracellular calcium flux assay

HMC3 cells were seeded at a density of 5.0 × 10^4^ cells/cm^2^ in a 96-well plate and incubated in a humidified atmosphere of 5% CO_2_ at 37^o^C overnight to adhere to the plate. The media was then removed, and the cells were washed with HEPES-buffered saline (HBS, pH 7.4) before the addition of fura-2 AM (5 µM) and Pluronic F-127 (0.05%) in HBS for 1 hour at room temperature in the dark. To allow for fura-2 AM de-esterification, the cells were washed twice with HBS before the addition of probenecid (2.5 mM) and BSA (0.1%) for 30 min at room temperature in the dark. Calcium flux was measured after treatment with histamine (100 µM) or non-fluorescent 4-bromo-A23187 (1 µM, Sigma) at 30 s via alternating fluorescent excitation wavelengths 340/380 at 510 nm emission at 3-second intervals over the course of 5 min on the BioTek Synergy H1 microplate reader (Agilent Technologies, Santa Clara, CA, USA). The results are presented as the ratio of 340/380 excitation wavelengths normalized to the first 30 s of reads prior to treatments.

## Fluorescence microscopy

HMC3 cells were seeded at a density of 5 × 10^4^ cells/cm^2^, grown on poly-D-lysine coated glass coverslips, and then treated with lipopolysaccharide (LPS, 1 µg/mL) or histamine (100 µM) for 24 hours. Following treatment, the cells were rinsed twice with PBS and fixed with 4% paraformaldehyde for 20 min at room temperature. For immunofluorescence staining, the cells were rinsed twice with PBS and permeabilized with 0.25% Triton X-100 for 15 min. After rinsing twice with PBS, nonspecific binding was blocked by incubating the cells with an Agilent X0909 serum-free protein block (Dako, Santa Clara, CA, USA) for 20 min at room temperature. The excess block was removed, and the cells were incubated in a humidified chamber overnight at 4ºC with anti-Iba-1 (Wako 019-19741) diluted 1:1000 in Dako antibody diluent (Agilent Dako) or anti-PrP^C^ POM2 IgG1κ (Millipore Sigma, Burlington, MA, USA) diluted 1:100 in Dako antibody diluent. After the cells were rinsed (2-min each), they were incubated with a goat anti-rabbit Alexa 568 secondary antibody (Invitrogen A11036) diluted 1:500 in Dako antibody diluent. Omission of primary antibody was used as a negative control. Following secondary incubation, the cells were rinsed three times with PBS and once with distilled water to remove salts and then mounted on Superfrost glass slides with ProLong Glass Antifade mounting media containing NucBlue (Invitrogen P36981). Images were acquired using an Olympus IX81 inverted microscope with a 20× objective and a Leica Stellaris 5 laser scanning confocal microscope with a 60× oil objective.

### Flow cytometric analysis of surface PrP^C^

HMC3 cells were seeded at a density of 5.0 × 10^4^ cells/cm^2^ in a 6-well plate and incubated in a humidified atmosphere of 5% CO_2_ at 37 ^o^C for 2 hours to adhere to the plate. The cells were then treated with histamine (10 µM-1000 µM) or LPS (1 µg/mL) and incubated for 6, 24, 48, or 72 hours at 37°C and 5% CO_2_. For experiments involving antagonists, cells were treated with 50, 100, or 500 µM of clemastine, ranitidine, JNJ-5207852, or JNJ-7777120 and incubated for 1 hour before treatment with 100 µM histamine. The cells were then detached with 0.25% trypsin-EDTA, washed with PBS, and blocked for 10 min on ice with 3% bovine serum albumin (BSA) to prevent nonspecific interactions (Marlborough, MA, USA) in PBS. Primary antibodies included mouse anti-PrP^C^ IgG1κ (POM2, Millipore Sigma) and mouse IgG1κ isotype antibodies (eBioscience). For unconjugated antibodies, cells were washed twice in 0.1% BSA in PBS and exposed to anti-mouse APC (Invitrogen) for 1 hour at 4°C on a plate shaker. The cells were then washed three times in 0.1% BSA in PBS and resuspended in 0.1% BSA in PBS before being analysed on a Cytoflex flow cytometer (Beckman Coulter). Cells and debris were separated by gating on an FSC-A vs. SSC-A plot while single cells were selected by gating on an FSC-A vs. FSC-H diagram. Changes in fluorescence were observed following gating.

### Western blot

HMC3 cells were seeded at a density of 5.0 × 10^5^ cells/mL in a 6-well plate and incubated in a humidified atmosphere of 5% CO_2_ at 37^o^C for 2 hours to adhere to the plate. The cells were then treated with histamine (10 µM-1000 µM) or LPS (1 µg/mL) and incubated for 24 hours at 37^o^C and 5% CO_2_. The cells were then removed with 0.25% trypsin-EDTA, washed with PBS, and lysed by resuspending the cell pellets in RIPA lysis buffer (100 mM Tris HCL pH 8.0, 10 mM EDTA pH 8.0, 100 mM NaCl, 0.5% sodium deoxycholate, 0.5% NP-40, EDTA-free cOmplete™ protease inhibitor cocktail (Roche), EDTA-free SIGMAFAST™ Protease Inhibitor Cocktail, and 6–10 U/mL benzonase (Millipore Sigma)). Human brain homogenates from patients with sporadic Creutzfeldt‒Jakob disease of the MM1 subtype or from healthy donors were used as positive controls. Bone marrow-derived mast cells from *Prnp* knockout mice were used as negative controls. After transfer to 0.2 μm PVDF membranes (Millipore Sigma), the membranes were blotted with anti-prion protein antibody, a.a. 109–112 (3F4, Millipore Sigma), or anti-histamine receptor antibodies: anti-HRH1 A82454 (antibodies.com), anti-HRH2 A83284 (antibodies.com), anti-HRH3 A98094 (antibodies.com), or anti-HRH4 A101422 (antibodies.com). After washing, the membranes were blotted with secondary antibodies diluted 1:15000: donkey anti-mouse 680RD (LI-COR) and donkey α-rabbit IRDYE800CW (LI-COR). All immunoblots in a single subfigure are from a single membrane that was stripped and re-probed between blots. The membranes were imaged with an Odyssey CLX Imaging System (LI-COR).

Western blot images were analysed using ImageJ (https://imagej.nih.gov/ij/). The images were inverted, and the brightness/contrast was adjusted by altering the minimum value. Band intensities were obtained from the original scan files using ImageJ2 version 2.14.0/1.54 F. To quantify band intensities, images were converted to grayscale and the background was subtracted before generating a ratio of the band with the protein of interest to the loading control.

### Statistical analysis

For flow cytometry experiments, three or more separate experiments were independently performed. For all other experiments, three or more separate experiments were independently performed, each containing at least three technical replicates. The standard error of the mean (SEM) or the standard deviation (SD) is indicated. The graphs were either plotted as bar graphs ± SEM or SD, or as violin plots with the median indicated via dotted line. *p-*values for statistical significance were determined by one-way ANOVA with Dunnett’s multiple comparisons or Tukey *post hoc* analysis for single treatments, *p* ≤ 0.05 (*), *p* ≤ 0.01 (**), *p* ≤ 0.001 (***), *p* ≤ 0.0001 (****). GraphPad Prism software version 9.5.1 was used to generate the figures and perform the statistical analysis (GraphPad Software, San Diego, CA, United States).

## Results

### Human-derived HMC3 microglia express histamine receptors

**Fig. 1:**
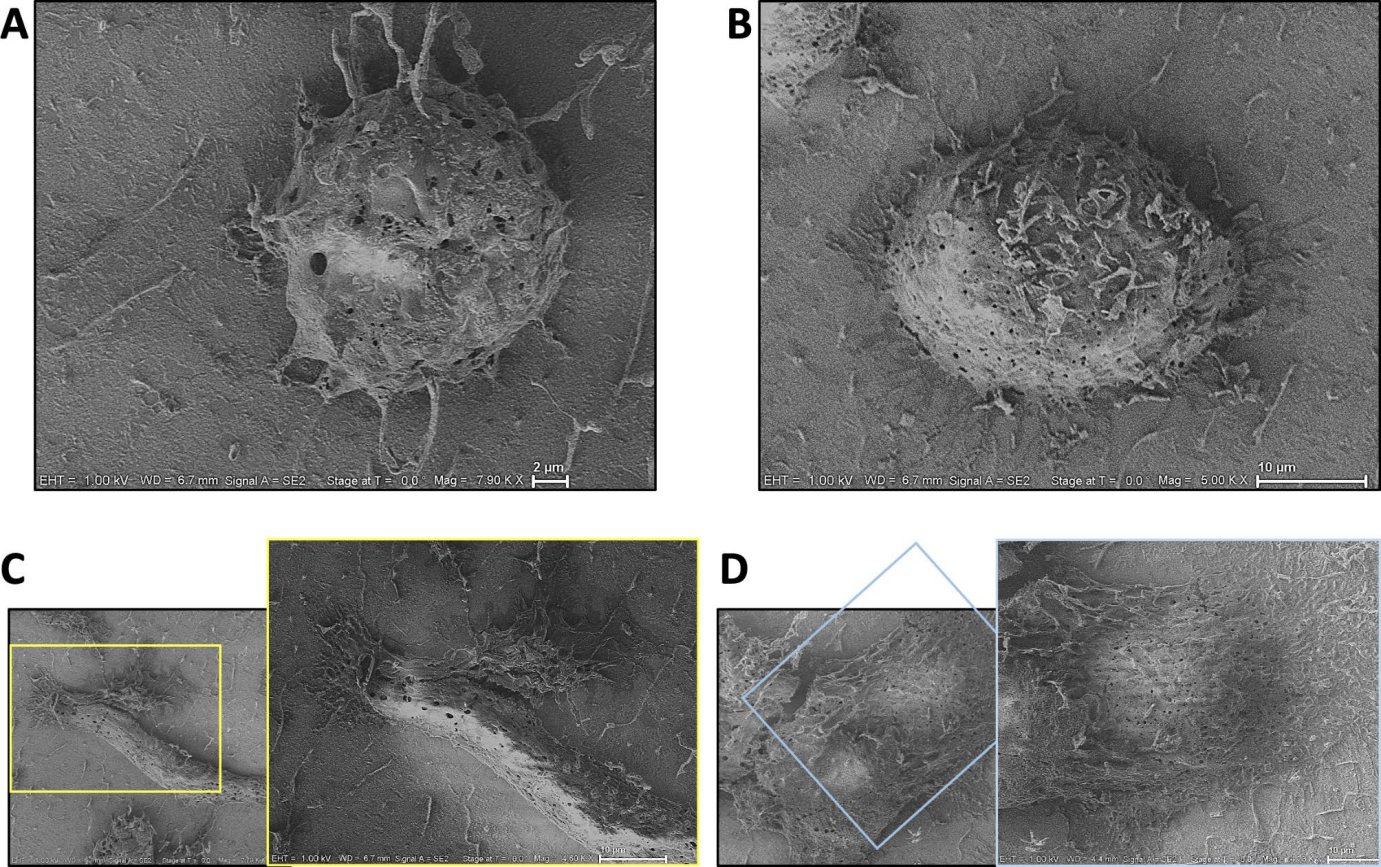
Cryo-SEM images of homeostatic HMC3 cells. HMC3 were cultured on coverslips treated with poly-D-lysine and Cryo-SEM images were collected. Cells were observed in various morphological states including (**A**) a homeostatic state, (**B**) an ameboid-like state, (**C**) a rod-like state, or (**D**) a ramified or hyper-ramified state. In bottom panels, boxes represent fields of view following increased magnification on the rod-like and ramified microglia.

Microglia are physiologically dynamic and modify their morphology actively^35^. Cryo-SEM of HMC3 cells show a heterogenous morphology with a very rough surface and several processes that appear to be pseudopodia (Fig. 1). Cells that interact or were in close proximity to each other appeared to have increased pseudopodia and we observed cells with various morphologies.

**Fig. 2:**
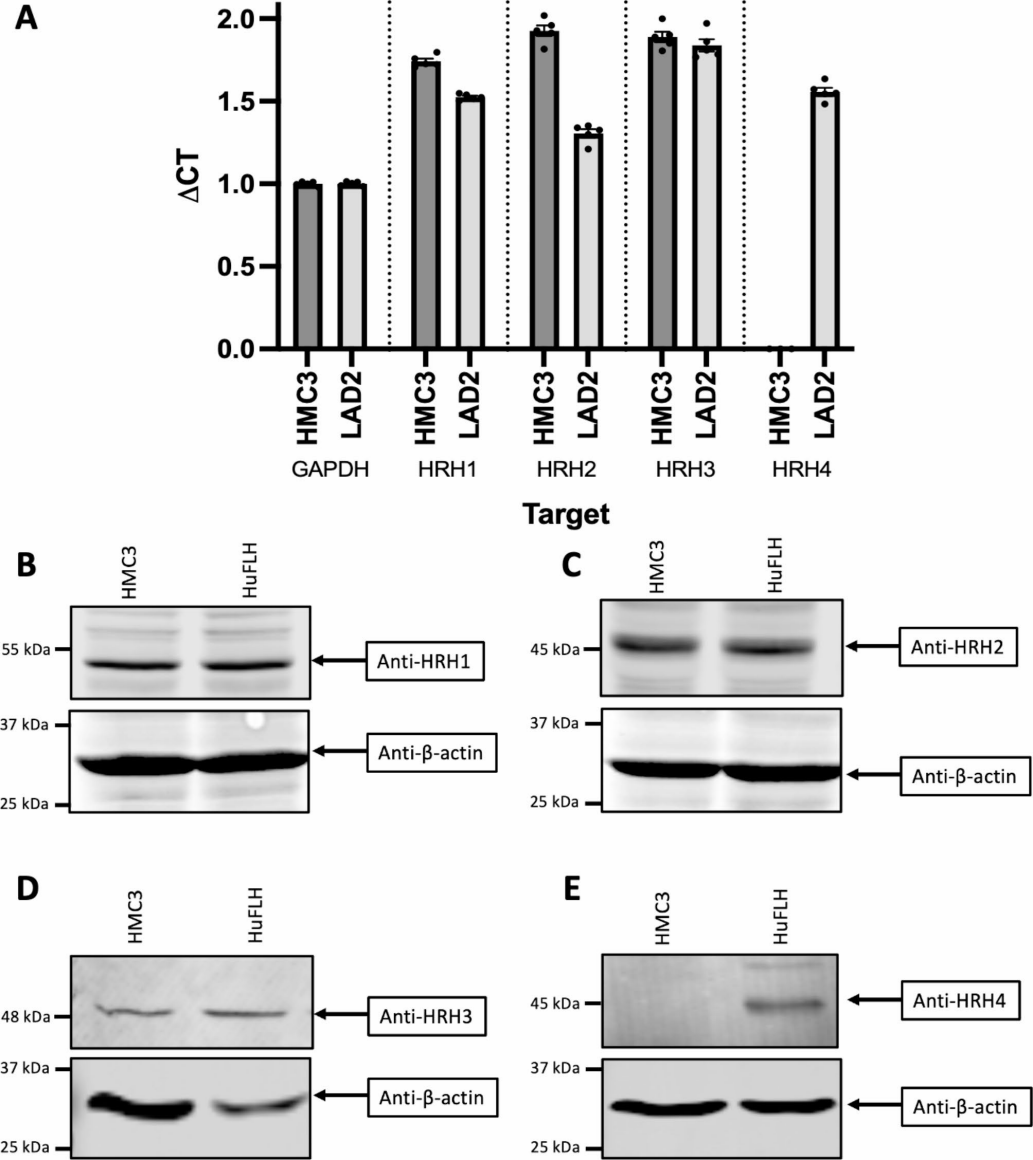
Human-derived HMC3 microglia express three of the four known histamine receptors. (**A**) RNA from HMC3 or LAD2 cells were isolated, and the expression of HRH1, HRH2, HRH3, and HRH4 was analysed. The data are presented as the average delta Ct (cycle threshold) for four independently isolated RNA samples; the expression of mRNA for each gene is relative to the GAPDH mRNA expression for each cDNA sample. (*N* = 5, bars indicate ± SD). (**B-E**) HMC3 or human frontal lobe homogenates (HuFLH) were isolated, and lysates were analysed via western blotting to test for protein expression of (**B**) HRH1, (**C**) HRH2, (**D**) HRH3, or (**E**) HRH4. Representative images of three independent experiments are shown. Uncropped blots are shown in Supplementary A.

There are four histamine receptors, each associated with different G proteins and with different signalling pathways dictating various functional outcomes^36^. To determine which of the four known histamine receptors were expressed by HMC3 microglia, HRH1, HRH2, HRH3, and HRH4 expression was analysed by qRT‒PCR. LAD2 cells were used as a positive control because these cells are known to express mRNA for all four histamine receptors^37^. HMC3 expressed HRH1, HRH2, and HRH3 mRNA but no HRH4 transcripts were detected (Fig. 2A). To measure the histamine receptor protein levels, we performed western blotting for the HRH1, HRH2, HRH3, and HRH4 receptors (Fig. 2B-E). Because the human frontal lobe contains all four histamine receptors^38,39^, we included human frontal lobe brain homogenates taken from healthy donors as positive controls. As expected, we detected measurable protein levels of HRH1, HRH2, and HRH3, but not HRH4 in HMC3 cells (Fig. 2B-E).

### Histamine stimulates HMC3 cells

**Fig. 3:**
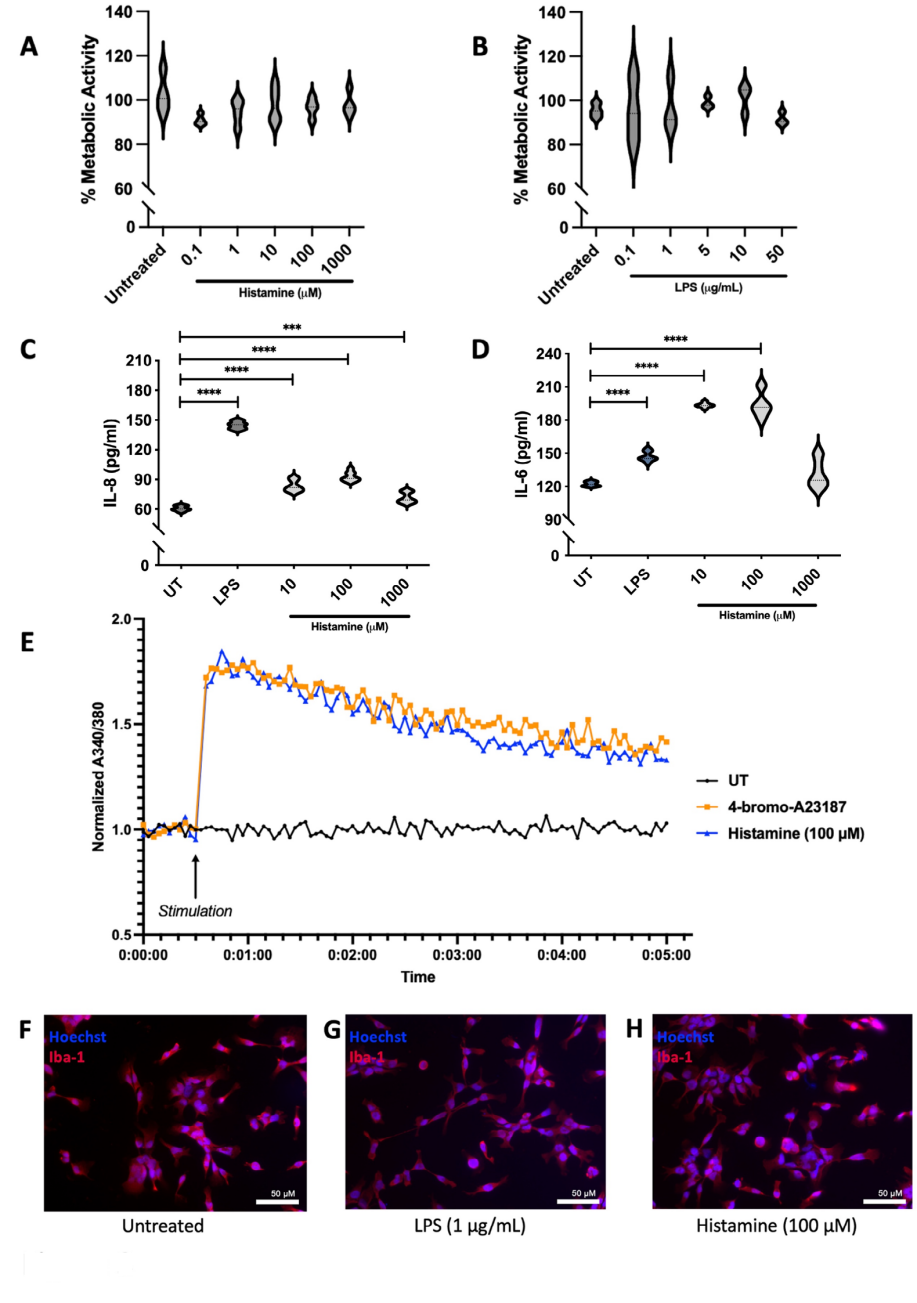
Histamine stimulates HMC3 cells to alter cytokine production and intracellular calcium levels, but not metabolic activity or Iba-1 expression. (**A**-**B**) HMC3 cells were stimulated with (**A**) histamine (0.1 µM – 1000 µM) or **(B)** LPS or left untreated for 24 hours and metabolic activity was measured by a reduction in XTT. The data are presented as the means ± SEMs (*N* = 6). (**C-D**) HMC3 cells were stimulated by histamine (10 µM, 100 µM, or 1000 µM), LPS (1 µg/mL), or left untreated for 24 hours, and (**C**) IL-8 and (**D**) IL-6 were measured by sandwich ELISA. Data are presented as the mean ± SEM (*N* = 9) and statistical significance was measured via one-way ANOVA and Dunnett’s multiple comparison post-hoc analysis relative to untreated (UT) cells. *p* ≤ 0.05 (*), *p* ≤ 0.01 (**), *p* ≤ 0.001 (***), *p* ≤ 0.0001 (****). (*N* = 4). (**E**) HMC3 cells were stimulated by histamine (100 µM) (blue) or 4-bromo-A23187 (1 µM) (orange) and intracellular calcium was measured via fura-2 AM dye which increases the 340/380 fluorescence ratio when bound to calcium ions. (**F-H**) Fluorescence microscopy images of HMC3 cells labelled with Hoechst 33342 (Hoechst) (blue) and Iba-1 (red) under (**F**) untreated conditions and following 24 hours treatment with (**G**) LPS and (**H**) histamine. Images are representative of four independent experiments using a 20× objective and a Leica Stellaris 5 microscope.

As the metabolic activity of microglia may be altered following stimulation, we tested potential metabolic changes in HMC3 microglia following histamine stimulation using a formazan reduction assay. This method utilizes XTT (2,3-bis(2-methoxy-4-nitro-5-sulfophenyl)-5-carboxanilide-2 H-tetrazolium), which is reduced by NADH produced by microglial mitochondria to form a soluble orange‒yellow formazan salt. Stimulation of HMC3 cells with histamine at various concentrations (0.1–1000 µM) did not alter the metabolic activity of these cells (Fig. 3A). We also demonstrated that stimulation of HMC3 cells with LPS at various concentrations (0.1–50 µg/mL) did not alter HMC3 metabolic activity (Fig. 3B**)**. To ensure that any observed alterations in the XTT assay were indeed due to metabolic changes within cells as opposed to changes in cell death or cell number, cellular viability and cell quantification were performed using a hemacytometer and we found no significant changes in cellular viability or cell quantity (Supplementary 2A-B).

In rodents, microglia respond to histamine by increasing the production of the inflammatory mediators IL-6^3^, TNF, IL-1β, and IL-10^10^. To test the response of HMC3 microglia to histamine, a neuroinflammatory mediator, we stimulated HMC3 cells with histamine and LPS and measured the release of the proinflammatory mediators IL-8 (Fig. 3C) and IL-6 (Fig. 3D) via sandwich ELISA. Histamine-induced stimulation of HMC3 cells increased the release of IL-8 at 10 µM, 100 µM, and 1000 µM (F (4,36) = 397.5, R^2^ = 0.9779, *p* = < 0.0001; 10 µM: 83.48 pg/mL, SEM ± 2.538, *p* = < 0.0001; 100 µM: 92.78 pg/mL, SEM ± 2.159, *p* = < 0.0001; 1000 µM: 71.65 pg/mL, SEM ± 2.381, *p* = 0.0008) relative to the release of IL-8 by the untreated controls (60.72 pg/mL). LPS also increased the amount of IL-8 (144.6 pg/mL SEM ± 1.767, *p* = < 0.0001) released from HMC3 cells compared to that released from untreated controls. Additionally, histamine-induced stimulation of HMC3 cells increased the release of IL-6 at 10 µM and 100 µM, but not 1000 µM (F (4,37) = 142.8, R^2^ = 0.9392, *p* = < 0.0001; 10 µM: 193.4 pg/mL, SEM ± 1.114, *p* = < 0.0001; 100 µM 193.9 pg/mL, SEM ± 4.628, *p* = < 0.0001; 1000 µM: 131.4 pg/mL, SEM ± 5.225, *p* = 0.2241) relative to the release of IL-6 by the untreated controls (122.2 pg/mL). LPS also increased the amount of IL-6 released from HMC3 cells (147.1 pg/mL SEM ± 1.114, *p* = < 0.0001) compared to that released from untreated controls. To substantiate our data, we also observed that HMC3 cells regulated the release of IL-8, IL-6, IL-10, IL-4, IFN-γ, and IL-12p70 release following histamine and LPS stimulation via electrochemiluminescence (Supplementary 3).

Intracellular calcium ions are highly regulated in microglia^40^ and changes in cytosolic calcium levels can be indicative of cellular stimulation. Our work demonstrated that, following histamine (100 µM) stimulation, HMC3 increased calcium influx resembling the changes in intracellular calcium observed in HMC3 cells stimulated with the calcium ionophore 4-bromo-A23187 **(**Fig. 3E**)**.

Iba-1 is a cytoplasmic protein that indicates microglial motility and is considered a microglial marker that may change expression levels following microglial stimulation^41,42^. We performed fluorescence microscopy to measure the expression of Iba-1 in HMC3 cells following histamine or LPS stimulation for 24 hours and found that these cells did not exhibit altered expression of this marker (Fig. 3F-H).

### HMC3 cells express PrP^C^

**Fig. 4:**
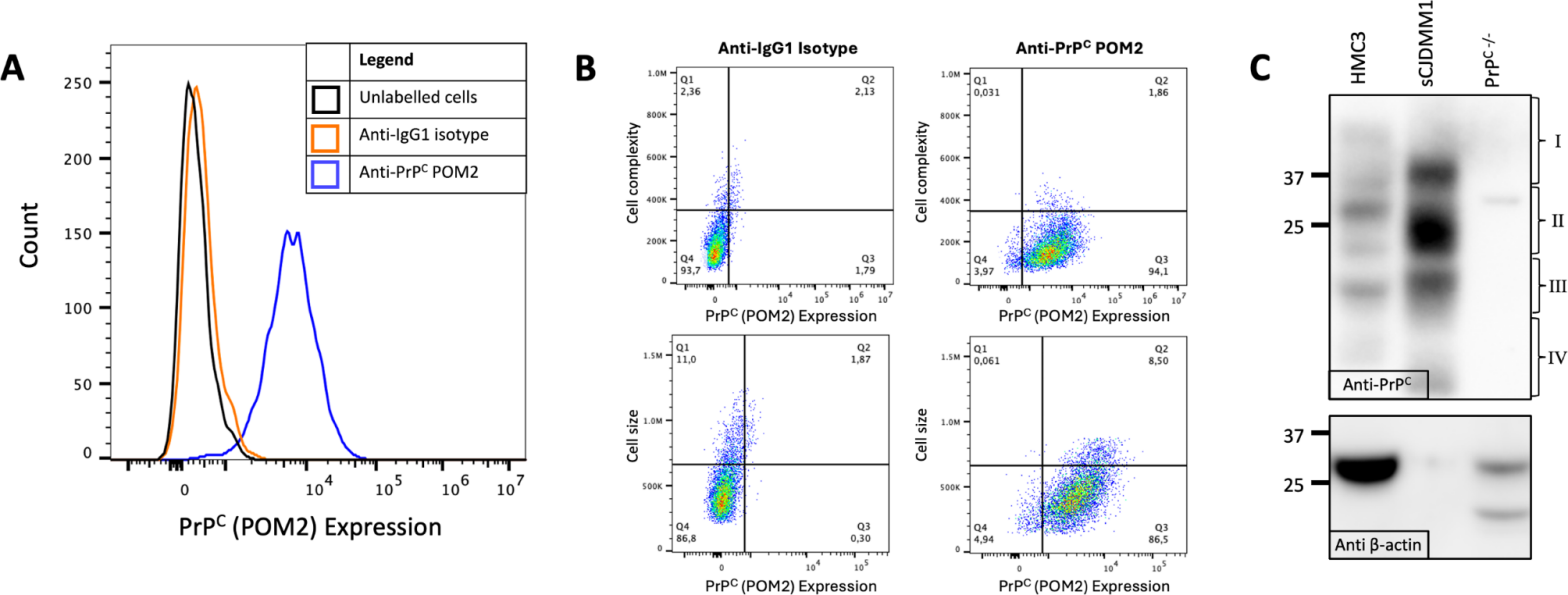
HMC3 cells express PrP^**C**^. (**A**) Flow cytometry immunolabelling of HMC3 cells with anti-PrP^C^ POM2. Black line: unlabelled HMC3 cells; orange line: HMC3 cells labelled with an IgG1κ isotype control; blue line: HMC3 cells labelled with the POM2 anti-prion protein antibody. (**B**) Flow cytometry scatterplots showing side scatter (cell complexity, top) or forward scatter (cell size, bottom) relative to PrP^C^ expression (x-axis). (**C**) Immunoblot of total PrP^C^ expression in HMC3 cells. Top: The anti-PrP^C^ 3F4 antibody indicated (I) di-, (II) mono-, and (III) unglycosylated forms, as well as (IV) PrP^C^ C-terminal fragments. sCJDMM1 from human brain homogenates was included as a positive control. PrP^C−/−^ BMMCs were included as negative control. Bottom: Anti-$\:{\upbeta\:}$-actin was used to indicate protein loading. (*N* = 3). Uncropped blots are shown in Supplementary B.

To test the expression of PrP^C^ by HMC3 microglia, we performed flow cytometry and western blotting. The expression of PrP^C^ by HMC3 microglia has not yet been described. We detected surface PrP^C^ via flow cytometry using an anti-PrP^C^ POM2 antibody at various concentrations (1 µg/mL to 10 µg/mL) (Fig. 4A-B**).** Total cellular PrP^C^ was measured via western blot (Fig. 4C**)**, where PrP^C^ appeared as several bands representing di-, mono-, and unglycosylated forms of the protein, as well as C-terminal fragments. Human brain homogenates taken from a patient with sporadic Creutzfeldt-Jakob disease of the MM1 subtype (sCJDMM1) were used as a positive control^33^. Of note, the variations in fragment sizes observed across the different sample types is due to different glycosylation profiles and cleavage products of the protein in these different cell types^43,44^. PrP^C^ is known to vary in its immunoblot banding appearance depending on cell type. Bone marrow-derived mast cells (BMMCs) from *Prnp* knockout mice, denoted as PrP^C−/−^, served as a negative control.

### Histamine stimulation alters HMC3 expression of surface PrP^C^

**Fig. 5:**
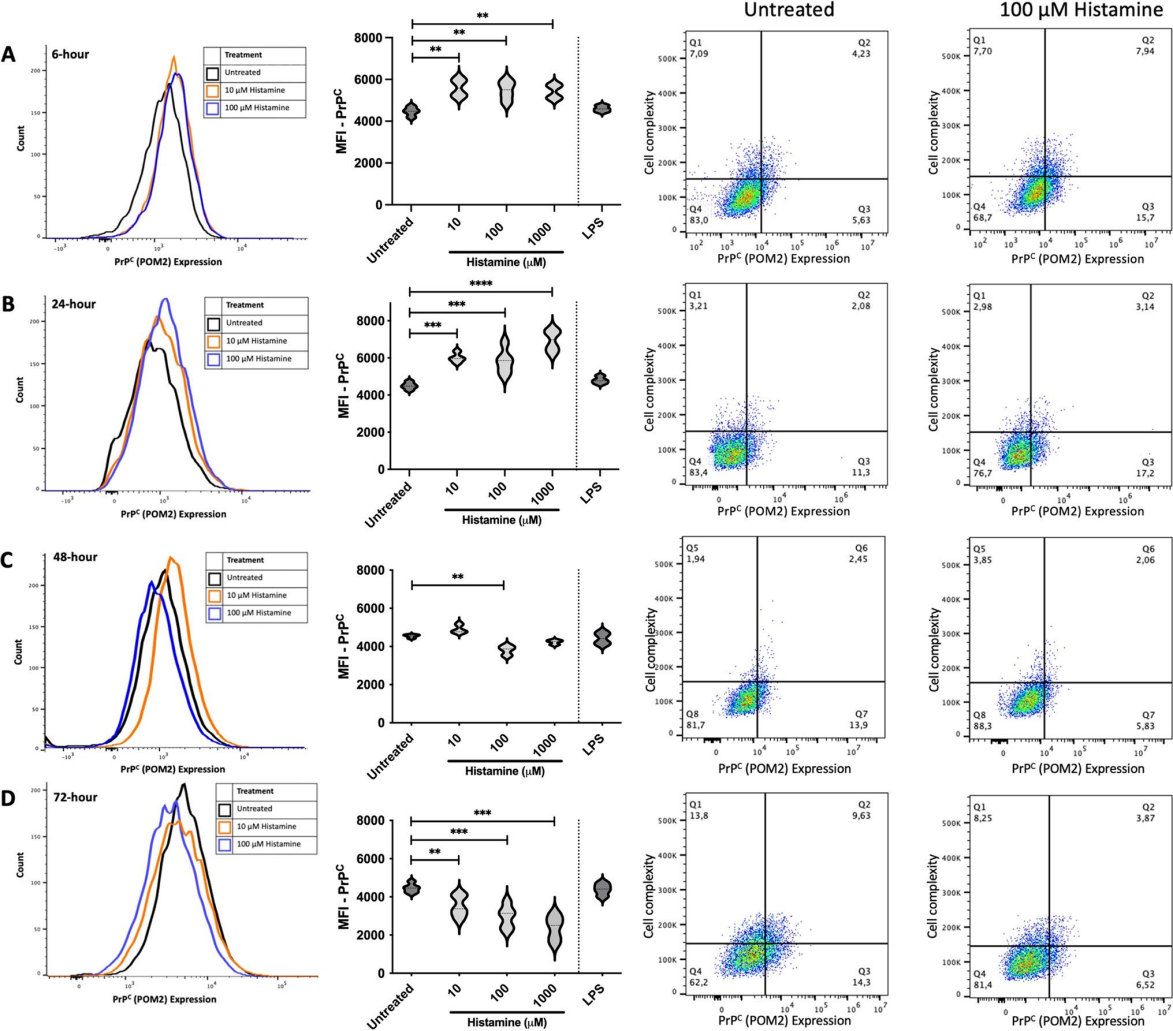
Histamine alters surface PrP^C^expression on HMC3 cells. Flow cytometry analysis of cell surface PrP^C^ following exposure to 10, 100, or 1000 µM histamine for (**A**) 6, (**B**) 24, (**C**) 48, or (**D**) 72 hours. MFI = Mean Fluorescent Intensity. Statistical significance was calculated using One-way ANOVA with Tukey’s post hoc analysis, *p* ≤ 0.01 (**), *p* ≤ 0.001 (***), *p* ≤ 0.0001 (****). Individual scatterplots were included to show side scatter against PrP^C^. Graphs plot side scatter (y-axis) as a measurement of cell complexity against PrP^C^ expression (x-axis). The percentages in each corner reflect the population of cells in each quadrant. (*N* = 4).

To measure how microglia alter their expression of PrP^C^ following stimulation with histamine, we measured surface protein and total protein by flow cytometry and western blot, respectively. Following 6 hours of stimulation, 10 µM, 100 µM, and 1000 µM histamine treatment led to similar increases in surface PrP^C^ (F (4,14) = 8.403, R^2^ = 0.7060, *p* = 0.0011; 10 µM mean MFI = 5594, SEM ± 239.9, *p* = 0.0018; 100 µM mean MFI = 5462, SEM ± 257.8, *p* = 0.0048; 1000 µM mean MFI = 5416, SEM ± 205.8, *p* = 0.0069) compared to those of the untreated controls (mean MFI = 4465) (Fig. 5A). Scatter plot analysis also showed that individual cells expressed an increase in surface PrP^C^ while slightly increasing complexity (Supplementary 4A) and size (Supplementary 5A). Following 24 hours of stimulation, 10 µM and 100 µM histamine treatment led to similar increases in surface PrP^C^ (F (4,17) = 21.47, R^2^ = 0.8348, *p* = < 0.0001; 10 µM mean MFI = 6009, SEM ± 148.1, *p* = 0.0003; 100 µM mean MFI = 5818, SEM ± 312.2, *p* = 0.0007), while 1000 µM led to the greatest increase (mean MFI = 6927, SEM ± 274.0, *p* = < 0.0001) compared to that of the untreated controls (mean MFI = 4498) (Fig. 5B). Scatter plot analysis also showed that individual cells expressed increased surface PrP^C^ and slightly increased cell complexity (Supplementary 4B) and size (Supplementary 5B).

To test the persistence of PrP^C^ changes over time following prolonged histamine exposure, we stimulated HMC3 microglia with histamine for 48 hours (F (4,12) = 10.15, R^2^ = 0.7719, *p* = 0.0008) and 72 hours (F (4,19) = 15.03, R^2^ = 0.7599, *p* = < 0.0001) and repeated these assays. At a 10 µM dose of histamine for 48 hours, HMC3 cells had similar levels of surface PrP^C^ expression (mean MFI = 4903, SEM ± 122.9, *p* = 0.1572) as the untreated controls (mean MFI = 4539) (Fig. 5C), whereas 72 hours of stimulation decreased surface PrP^C^ (mean MFI = 3508, SEM ± 274.2, *p* = 0.0273) relative to that of the untreated controls (mean MFI = 4458) (Fig. 5D). At a 100 µM dose of histamine for 48 and 72 hours, HMC3 cells had significantly decreased surface PrP^C^ (mean MFI = 3809, SEM ± 134.8, *p* = 0.0034, untreated mean MFI = 4539; mean MFI = 2951, SEM ± 288.5, *p* = 0.0006, untreated mean MFI = 4458, respectively). Similarly, at a 1000 µM dose of histamine, HMC3 slightly decreased surface PrP^C^ (mean MFI = 4227, SEM ± 78.01, *p* = 0.2537, untreated mean MFI = 4539) following 48 hours of stimulation and significantly decreased surface PrP^C^ (mean MFI = 2383, SEM ± 305.8, *p* = < 0.0001, untreated mean MFI = 4458) following 72 hours of stimulation. Scatter plot analysis also revealed that individual cells exhibited a concentration-dependent decrease in surface PrP^C^ without altering complexity following 48 and 72 hours of histamine stimulation (Supplementary 4C-D) or size (Supplementary 5C-D).

To assess whether decreases in PrP^C^ over time were due to prolonged culture conditions, we plotted PrP^C^ surface protein expression over time and compared its expression levels with those of untreated cells (Supplementary 6). We found that in untreated HMC3 cells, PrP^C^ expression did not significantly change over time (Supplementary 6A), whereas in response to histamine stimulation, PrP^C^ expression first increased, then decreased over extended exposure times (Supplementary 6B-D).

**Fig. 6:**
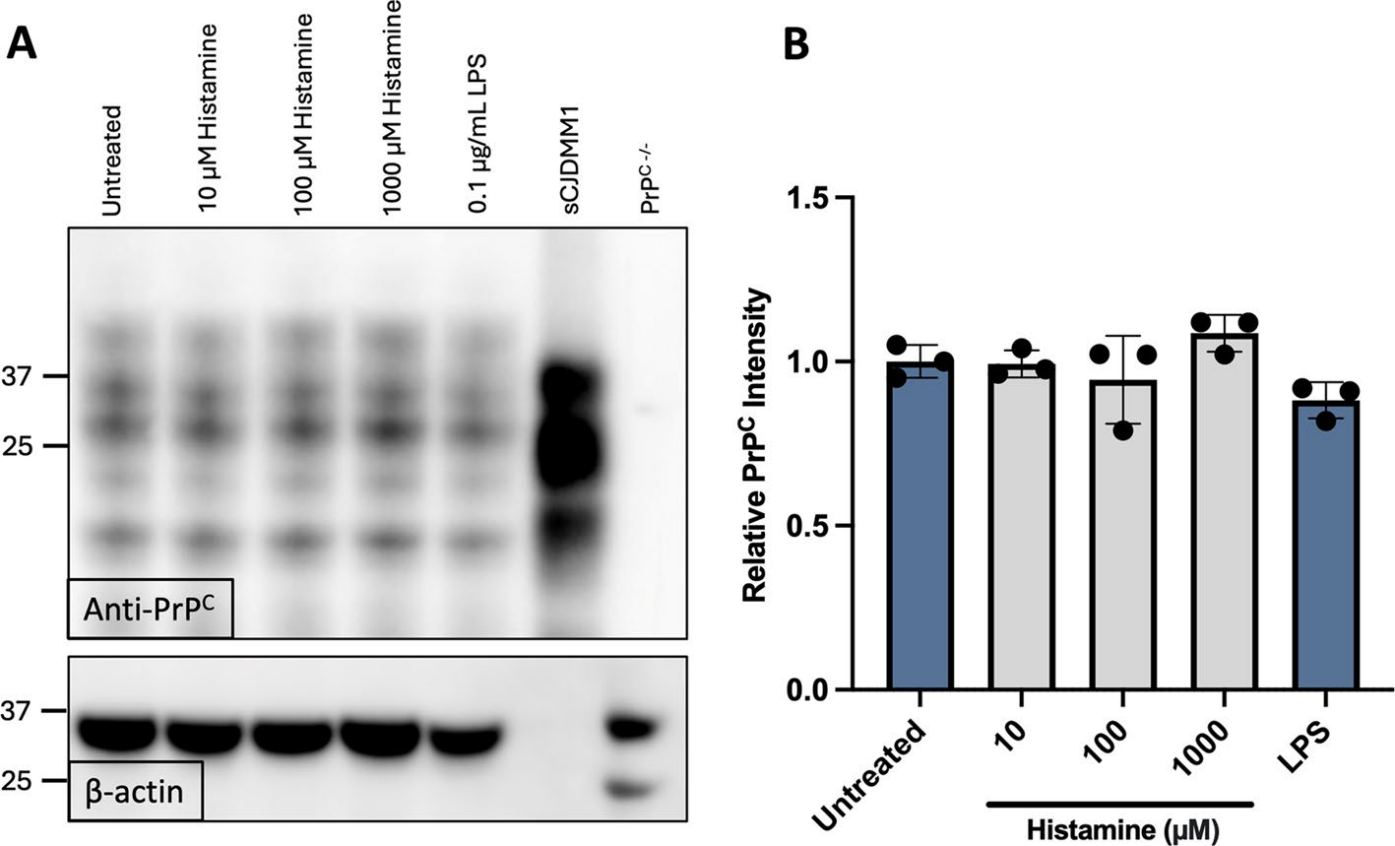
Histamine does not change total PrP^C^expression in HMC3 cells. (**A**) Representative western blot image of HMC3 cells treated with various concentrations of histamine or LPS for 24 hours. *Top*: Anti-PrP^C^ 3F4 antibody. *Bottom*: Anti-$\:{\upbeta\:}$-actin was used to indicate protein loading. Human sCJDMM1 was included as a positive control. PrP^−/−^ BMMC were included as negative control. (**B**) Quantification of western blot band intensity. The relative intensity was quantified against that of untreated controls. (*N* = 3 bars indicate ± SEM). Uncropped blots are shown in Supplementary C.

We next tested whether the changes in surface PrP^C^ levels were due to changes in the total amount of cellular PrP^C^ present or changes in the localization of PrP^C^. HMC3 cells were treated with histamine for 24 hours before they were lysed, and total PrP^C^ was quantified via western blotting. We found that total PrP^C^ expression was not significantly altered following histamine or LPS stimulation (Fig. 6A-B), indicating that changes in surface PrP^C^ expression were likely due to changes in PrP^C^ localization on the cell surface.

### Fluorescence microscopy of HMC3 PrP^C^ expression

**Fig. 7:**
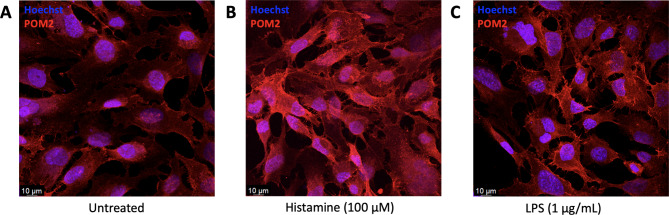
Immunofluorescence detection of surface PrP^C^expression in HMC3 cells treated with histamine and LPS. Representative fluorescence microscopy images of HMC3 labelled with Hoechst 33342 (blue) and anti-PrP^C^ POM2 (red) under (**A**) untreated conditions or after 24 hours of stimulation by (**B**) histamine or (**C**) LPS. Images are representative of three independent experiments using a 20× objective and a Leica Stellaris 5 microscope.

To further visualize any changes in PrP^C^ surface localization in HMC3 cells, we used fluorescence microscopy to assess the surface expression of PrP^C^ following histamine and LPS stimulation using mouse monoclonal anti-PrP^C^ POM2 antibody. HMC3 cells stimulated with histamine and LPS exhibited notable increases in the expression of PrP^C^(Fig. 7B and C**)** relative to untreated cells (Fig. 7A). Specifically, HMC3 cells stimulated with histamine increased PrP^C^ expression slightly more than cells stimulated with LPS. Neither histamine or LPS treatment appeared to modify HMC3 morphology.

### Histamine-induced changes in surface PrP^C^ are due to signalling by the HRH2 receptor

**Fig. 8:**
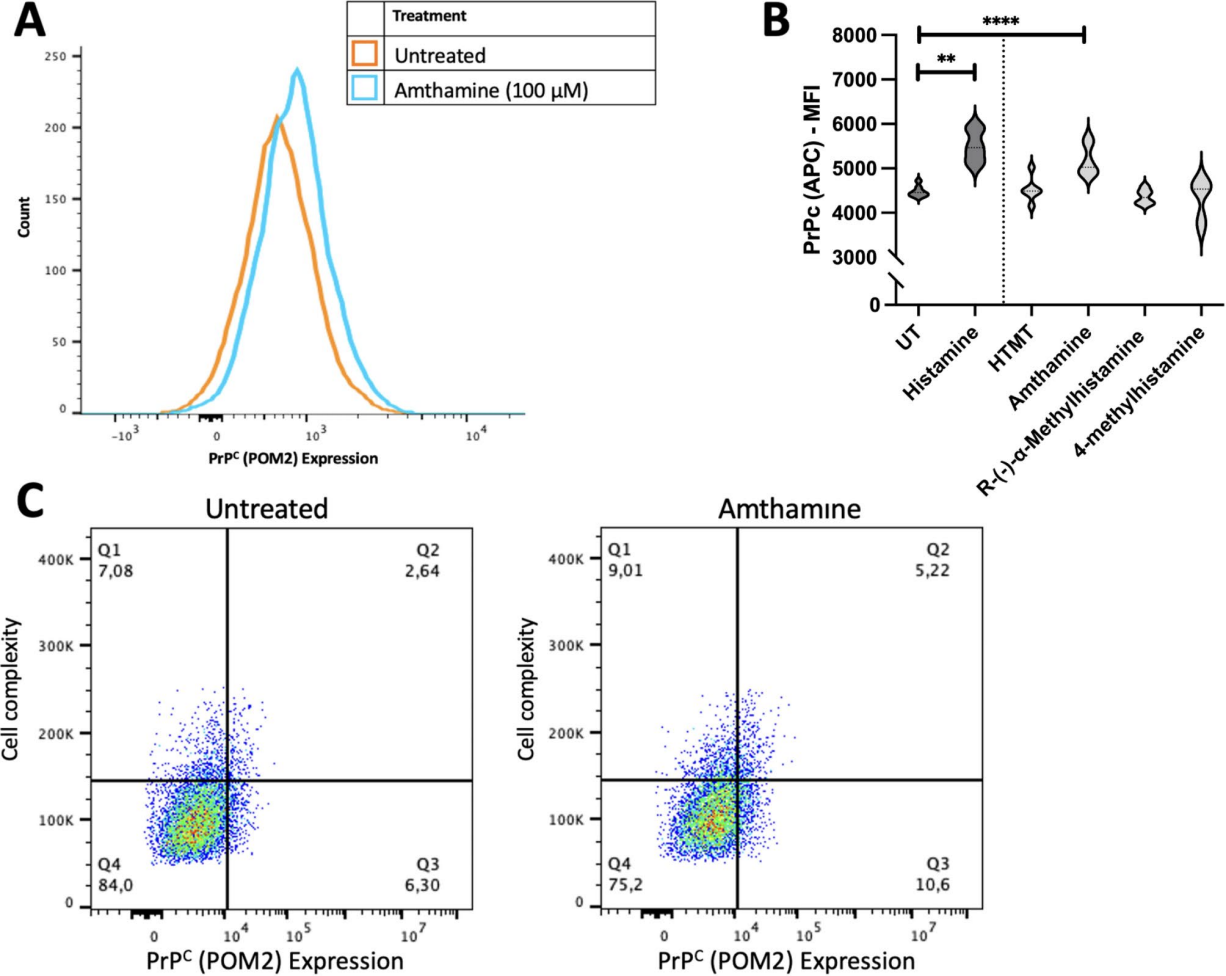
PrP^C^expression following activation of histamine receptors. (**A**) HMC3 cells were stimulated with the HRH2 agonist amthamine for 24 hours and surface PrP^C^ levels were measured via flow cytometry. (**B**) HMC3 cells were treated with 100 µM of histamine, HTMT (an HRH1 agonist), amthamine (an HRH2 agonist), R-(–)-α-methylhistamine (an HRH3 agonist), or 4-methylhistamine (an HRH4 agonist) for 24 hours and surface PrP^C^ levels were measured via flow cytometry. The mean fluorescent intensity (MFI) was quantified, and statistical significance was calculated using one-way ANOVA with Tukey’s post hoc analysis, *p* ≤ 0.01 (**), *p* ≤ 0.0001 (****). (**C**) Individual scatterplots show side scatter against PrP^C^. Graphs plot side scatter (y-axis) as a measurement of cell complexity against PrP^C^ expression (x-axis). The percentages in each corner reflect population of cells in each quadrant. (*N* = 4).

Having demonstrated that 24-hour histamine stimulation increases surface PrP^C^ expression, we next investigated which histamine receptor may be responsible for these changes. Accordingly, we treated HMC3 cells with histamine receptor agonists for 24 hours: we used 100 µM each of HTMT, Amthamine, R-(—)-α-methylhistamine, or 4-methylhistamine which activate HRH1, HRH2, HRH3, or HRH4, respectively. We then measured surface PrP^C^ levels via flow cytometry (Fig. 8) and demonstrated that activation of the HRH2 receptor increased PrP^C^ expression (F (5,31) = 15.43, R^2^ = 0.7133, *p* = < 0.0001; mean MFI = 5064, SEM ± 156.8, *p* = 0.0012) relative to that in untreated controls (mean MFI = 4511).

To determine whether the HRH2 receptor was the sole contributor to changes in PrP^C^ expression, we treated HMC3 cells with histamine receptor antagonists for 1 hour prior to stimulating them with 100 µM histamine and repeated experiments to measure PrP^C^ expression changes and cytokine release. We first measured cellular viability and cell proliferation following treatments of 50, 100, or 500 µM of histamine receptor antagonists. We used clemastine, ranitidine, JNJ-5207852, and JNJ-7777120 to block HRH1, HRH2, HRH3, and HRH4, respectively. Using a hemacytometer, we indicated that HMC3 cells treated with 50 or 100 µM of each of these compounds did not significantly alter their cellular viability (Supplementary 7A-C) or proliferation (Supplementary 7E-G), whereas 500 µM significantly decreased both viability (Supplementary 7D) and cell proliferation (Supplementary 7H).

**Fig. 9:**
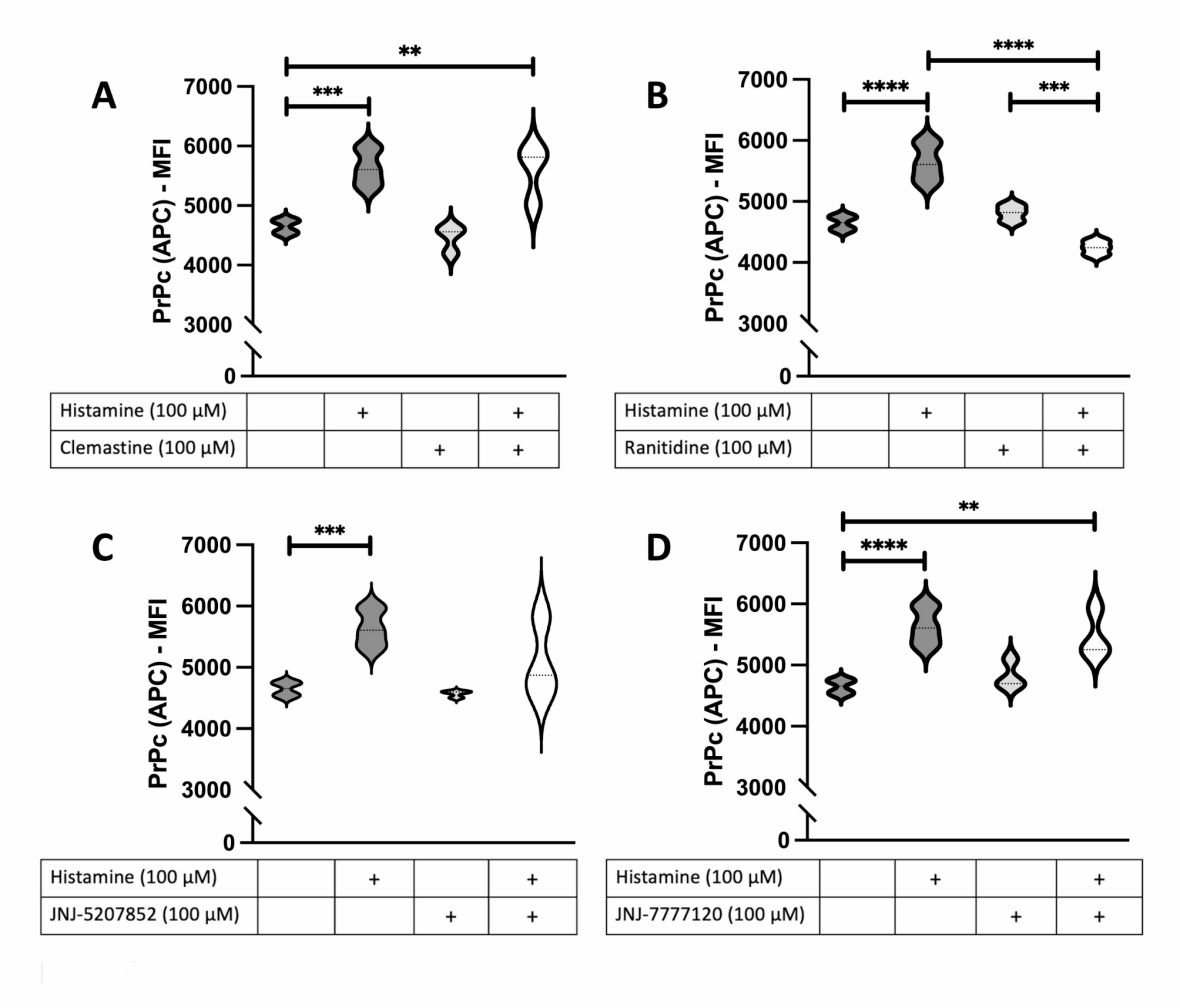
Histamine acts via the HRH2 receptor to upregulate surface PrP^C^expression. HMC3 cells were treated with 100 µM (**A**) clemastine (an HRH1 antagonist), (**B**) ranitidine (an HRH2 antagonist), (**C**) JNJ-5207852 (an HRH3 antagonist), or (**D**) JNJ-7777120 (an HRH4 antagonist) for 1 hour, and then treated with 100 µM histamine for 24 hours, after which PrP^C^ expression was measured by flow cytometry. Statistical significance was calculated using one-way ANOVA with Tukey’s post hoc analysis, *p* ≤ 0.01 (**), *p* ≤ 0.001 (***), *p* ≤ 0.0001 (****). (*N* = 4).

Accordingly, we then measured surface PrP^C^ via flow cytometry following 1 hour of 100 µM histamine receptor antagonism treatments and 24 hours of 100 µM histamine-induced stimulation (Fig. 9). As expected, blocking the HRH2 receptor prior to histamine treatment prevented the increase in surface PrP^C^ (F (3,14) = 40.95, R^2^ = 0.8977, *p* = < 0.0001; mean MFI = 4242, SEM ± 180.6, *p* = < 0.0001; histamine treatment alone: mean MFI = 5635). In contrast, the inhibition of HRH1, HRH3, and HRH4 did not prevent the histamine-induced changes in surface PrP^C^ expression HRH1 inhibition: (F (3,14) = 21.15, R^2^ = 0.8193, *p* = < 0.0001, mean MFI = 5579, SEM ± 253.5, *p* = 0.9914; HRH3 inhibition: (F (3,14) = 12.96, R^2^ = 0.7353, *p* = < 0.0001, mean MFI = 5092, SEM ± 301.8, *p* = 0.1082; HRH4 inhibition: (F (3,14) = 18.09, R^2^ = 0.7950, *p* = < 0.0001, mean MFI = 5477, SEM ± 230.2, *p* = 0.8195; histamine treatment alone: mean MFI = 5635). Scatter plot analysis supported these data (Supplementary 8), and these results support HRH2 as the receptor likely involved in the observed surface PrP^C^ expression changes.

We additionally measured PrP^C^ surface expression following 1-hour treatments of 50 and 500 µM histamine receptor antagonism followed by 24 hours of histamine treatment to compare these results with our 100 µM antagonist results and create a dose-response curve of various doses of histamine receptor antagonist compared to surface PrP^C^ expression (Supplementary 9). These results highlighted that 100 µM of ranitidine antagonism of HRH2 was sufficient to block histamine-induced increases in PrP^C^ surface expression and increased doses of histamine receptor antagonists caused various changes in PrP^C^ expression, likely due to aforementioned changes in cellular viability and proliferation.

Following confirmation that HRH2 was the primary receptor involved in PrP^C^ expression changes, we next tested whether the HRH2 receptor was responsible for the histamine-induced changes in cytokine release. After a 1-hour pretreatment with the histamine receptor antagonists, we stimulated HMC3 with 100 µM histamine and measured IL-8 and IL-6 release (Supplementary 10). We found that histamine-induced IL-8 released following blocking of HRH1 (91.33 pg/mL, SEM ± 4.024, R^2^ = 0.04584, *p* = 0.5802), HRH2 (80.00 pg/mL, SEM ± 4.140, R^2^ = 0.4030, *p* = 0.0663), HRH3 (90.33 pg/mL, SEM ± 3.823, R^2^ = 0.01708, *p* = 0.7376), and HRH4 (94.67 pg/mL, SEM ± 4.041, R^2^ = 0.2193, *p* = 0.2036) was not significantly different from that of cells treated with histamine alone (89.00 pg/mL). Interestingly, histamine-induced IL-6 release following blocking of HRH2 (165.3 pg/mL, SEM ± 7.189, R^2^ = 0.6109, *p* = 0.0128) was significantly lower than that of cells treated with histamine alone (189.2 pg/mL), whereas histamine-induced changes in IL-6 following inhibition of HRH1 (185.7 pg/mL, SEM ± 7.452, R^2^ = 0.03055, *p* = 0.6529), HRH3 (187.3 pg/mL, SEM ± 8.069, R^2^ = 0.007321, *p* = 0.8268), and HRH4 (192.3 pg/mL, SEM ± 7.762, R^2^ = 0.02322, *p* = 0.6955) was not significantly different from that of histamine treatment alone (189.2 pg/mL).

## Discussion

In this study, we asked whether the cellular prion protein would (PrP^C^) play a role in modulating microglial responses to stimuli, with a particular focus on histamine as a stimulus. To our knowledge, this work is the first to demonstrate that histamine stimulates the human-derived microglial cell line HMC3, leading to the release of proinflammatory cytokines and increases in intracellular calcium ions but no significant changes in metabolic activity. Additionally, we are the first to show that HMC3 cells express PrP^C^, and following histamine stimulation, these cells alter the surface levels but not the total expression of PrP^C^ via stimulation of the HRH2 receptor.

HMC3 cells were utilized for this study and are admittedly an imperfect model for primary human brain-derived microglial cells. Currently, there are limited options for human microglial cell lines available for in vitro research. HMC3 have been utilized in proinflammatory conditions and this is frequently used in both healthy and neurodegenerative disease models^34,45–48^. HMC3 are physiologically similar to primary microglia because they express numerous biomarkers of microglia and macrophages such as CD11b, CD45, CD68, and Iba-1^48^.

Our team and others have previously demonstrated that HMC3 cells modify cytokine production following stimulation via various mechanisms and treatments^34,49^. Our current data show that histamine stimulation alters microglial release of IL-8 and IL-6, two key inflammatory mediators involved in neuroinflammation and microglial inflammatory signalling^50,51^. IL-8 is a chemokine largely involved in immune cell regulation, and this chemokine plays a key role in cell signalling as it recruits neutrophils and various other immune and inflammatory cells, such as monocytes and macrophages, to sites of tissue damage and infection in the CNS^52,53^. Walker et al. indicated that this inflammatory mediator is the most highly upregulated factor following Aβ_1–42_-induced stimulation of human microglia, highlighting the importance of this chemokine as a neuroinflammatory mediator^54^. Notably, as microglia express the IL-8 receptors CXCR1^55^ and CX3CR1^56^, which are known to be involved in the recruitment of microglia to sites of neuroinflammation, the release of IL-8 by stimulated microglia in the brain may further promote neuroinflammation and the accumulation of additional immune cells. On the other hand, IL-6 is produced by various cells in the CNS, and it plays an important role in both homeostatic states^57^ and in states of tissue injury and neuroinflammation^58^. IL-6 is a key indicator of microglial stimulation^59^, as it is thought to play an important role in neuroinflammatory signalling. Studies have indicated that following stimulation of primary cultures of human CNS cells, microglial IL-6 release is precisely and dynamically modified^57,60^. Our data showed that histamine induces human microglia to dynamically increase IL-8 and IL-6 levels, further suggesting that histamine is a key regulator of neuroinflammatory signalling in microglia.

Studies by Xia et al., Rocha et al., and Zhang et al. have indicated that murine microglia respond to histamine by increasing intracellular Ca^2+^ and phagocytosis^10,15,17^. Our data showed that histamine altered calcium flux in HMC3 cells similarly to that of 4-bromo-A23187, a calcium ionophore that mediates the transport of calcium ions intracellularlly^61^. Previous studies have suggested that stimulated microglia alter intracellular calcium as a means to regulate cellular proliferation, cellular motility, ramification, and the release of proinflammatory cytokines^62^.

Our data indicated that histamine may modify how microglia produce inflammatory mediators. This work demonstrated that histamine-induced stimulation did not alter the expression of Iba-1. This molecule is a cytoplasmic protein that is typically used to identify microglia and macrophages; however, there is conflicting evidence regarding whether Iba-1 levels are altered following microglial stimulation^41,42,63^. Furthermore, recent research on microglia and histamine has demonstrated that histamine-induced stimulation of murine microglia alters motility and modifies the release of the proinflammatory cytokines IL-1β and TNF^13^, increases microglial ROS production^15^ and modifies the release of proinflammatory mediators by these cells^10^. These studies substantiated our electrochemiluminescence data which demonstrated that microglia altered their release of IL-8, IL-6, IL-10, IL-4, IFN-γ, and IL-12p70 following histamine- and LPS-induced stimulation, although a more comprehensive analysis of mediator release is necessary to fully understand the transcriptional pathways that are engaged and the regulatory networks that may control how these mediators are secreted.

Previous studies in murine models have indicated ambiguity in the expression of histamine receptors in murine models, with some studies suggesting that murine microglial cell lines express all four known histamine receptors (HRH1, HRH2, HRH3, and HRH4)^13^, while others suggest that primary murine models only express certain receptors on certain cell types^18,64^. There has been little research on histamine receptor expression in human microglia. To the best of our knowledge, we are the first to show that human-derived microglia express HRH1, HRH2, and HRH3 mRNA and protein, but not HRH4. The absence of HRH4 in HMC3 cells in the present study concurs with previously reported transcriptomic and proteomic data, which indicates that there is limited expression of HRH4 in the central nervous system^17,65^, which is typically found in neurons in the deep laminae of the cortex in humans^38^.

There is growing evidence for the role of PrP^C^ in the immune system, and this protein is variably expressed by numerous immune cells, including haemopoietic stem cells^66^, dendritic cells, T cells^67^, and leukocytes^68^, suggesting that physiological PrP^C^ may be linked to inflammatory and immune responses. Murine microglia express PrP^C^, and Shi et al. demonstrated that this protein is likely involved in the maintenance of microglia in homeostatic and reactive states^20^. Although many studies have indicated that microglia are involved in prion disease^69–71^, very few studies have investigated PrP^C^ expression in human microglia; we are one of the first to directly show PrP^C^ protein expression in human-derived microglia.

Microglial stimulation induces changes in the expression of genes and proteins involved in the immune response and activation^72,73^. Although PrP^C^ expression on activated murine microglia has been examined^74^, our data are the first to suggest that human microglial stimulation may alter cell-surface PrP^C^ expression, suggesting that PrP^C^ may function as a mediator of inflammation and the immune response in the human brain. Our data showing that PrP^C^ expression is altered in a time- and concentration-dependent manner support the findings of Izzy et al., who suggested that unique changes in cellular protein expression occur at different time points following modifications in microglial microenvironments^75^. More specifically, Izzy et al. reported that microglia alter inflammatory gene expression in a time-dependent manner following cerebral contusion in mice, leading to changes in microglial sensitivity to tissue damage. Our data support this concept of dynamic changes in microglial inflammatory gene and protein expression following stimulation of these cells. Our work expands upon this evidence of histamine-induced changes in PrP^C^ by demonstrating that this stimulation is primarily induced by HRH2-receptor signalling.

The four histamine receptors are GPCR receptors, characterized by their seven transmembrane α-helices, three intracellular loops, and three extracellular loops^76^. In the CNS, GPCRs have the highest expression of any receptor type^77^, and these receptors modulate the CNS to precisely and dynamically respond to endogenous and exogenous stimuli^78^. Our data indicated that HRH2 was the primary receptor responsible for histamine-induced changes in PrP^C^ surface expression in microglia, however this inhibition of histamine-induced changes in PrP^C^ did not block IL-8 release and only partially blocked IL-6 release. This is an interesting observation, and these results suggest that modulation of surface PrP^C^ is dynamic and receptor-specific as HRH2 does not appear to be involved in cytokine release. The HRH2 receptor acts via the Gαs subunit which promotes 3’-5’-cyclic adenosine monophosphate (cAMP) generation^79^. cAMP signalling via the phosphoinositide 3-kinase/AKT (PI3K/AKT) pathway is crucial for inflammatory signalling in Alzheimer’s and other neurodegenerative diseases^79^, and this cellular messenger is a key regulator of microglial function and stimulation^80^. Notably, cAMP signalling tightly regulates calcium levels and dynamics in neuronal cells and astrocytes^81^, and accordingly, the changes in calcium flux observed in the present study may indeed support the notion of HRH2 stimulation via this mechanism. Additionally, histamine and its receptors are known to promote and regulate wakefulness^82^, and PrP^C^ is also thought to be crucial for circadian rhythms and sleep^83^. Since our data highlights the dynamic regulation of PrP^C^ and histamine, it is possible that histamine receptors may regulate the accumulation and stimulation of microglia at sites of inflammation, promoting an increase in inflammatory cytokines. The exact role of PrP^C^ as a neuroinflammatory regulator is not clear, but this protein may be involved in facilitating this intercellular communication and regulating the release of cytokines at the microglial membrane^84^. The expression of surface PrP^C^ appears to be linked to cytokine production and secretion in other cells^23,85^, suggesting that this link between secretion and PrP^C^ shuttling may be a common phenomenon. As PrP^C^ has previously been identified as a scaffolding adaptor for other receptors^43,86^, our data suggests that PrP^C^ may localize to the cell surface to promote cytokine release immediately following histamine stimulation. However, as PrP^C^ gradually decreases following extended histamine stimulation, it may in turn gradually decrease cytokine production thereby attenuating the inflammatory response. In this way, PrP^C^ may act as a control dial that tightly modulate microglial function. Although the molecular mechanisms are unclear, when this balance is interrupted, chronic inflammation may occur, and accordingly, this mechanism may prove as a useful drug target in treating neuroinflammation.

PrP^C^ is a membrane-bound protein typically located on the cell surface^87^ where it regulates cellular transport and participates in cellular signalling^88^. The protein has been found in pre- and postsynaptic compartments of nerve terminals^89^, the outer membrane of neurons^90^, and the cytoplasm and nucleus of both neurons and glial cells of the CNS^88,91,92^. The localization of this protein is dynamic, and the protein is known to change location in neurodegenerative disease^93^. Our microscopy results demonstrated that following 24 hours of stimulation by both histamine and LPS microglia modify the localization of PrP^C^, which is trafficked to the cell surface, depending on the duration of stimulation. These microscopy data are particularly interesting as our flow cytometry results demonstrated that histamine stimulation, but not LPS stimulation, increased surface PrP^C^. These discrepancies may be due to differences in signalling pathways; whereas LPS stimulates the TLR4 receptor^94^, histamine acts via G-coupled protein receptors^95^. As PrP^C^ varies in localization in different cellular compartments depending on various physiological conditions^93,96,97^, these differences in histamine- and LPS-induced stimulation may highlight the separate, distinct signalling pathways that induce microglial regulation of PrP^C^ expression. Though the molecular pathway remains unclear, our data highlighting the different mechanisms responsible for discrepancies in histamine- and LPS-induced stimulation of microglia could prove a useful pharmacological target in treating and regulating neuroinflammation.

Taken together, these data suggest that histamine stimulates human-derived microglia to increase the release of inflammatory cytokines, increase intracellular calcium levels, and alter PrP^C^ surface expression, likely via the HRH2 receptor. Future studies are needed to further understand the role of histamine and PrP^C^ in inflammatory signalling in human models. Specifically, experiments using primary human microglia should be conducted and tests on physiologically relevant microglial mediators such as P2RY12 should be performed, as others have shown that microglial responses to histamine are primarily dependent on this receptor in microglia^17^.

## Electronic supplementary material

Below is the link to the electronic supplementary material.

Supplementary Material 1

Supplementary Material 2

## Data Availability

All data generated or analysed during this study are included in this published article [and its supplementary information files].

## Abbreviations

CNS: Central nervous system
Cryo-SEM: Cryo-scanning electron microscopy
ELISA: Enzyme-linked immunosorbent assay
FSC: Forward-scatter (cell size measurement)
HMC3: Human microglia clone 3
LAD2: Laboratory of allergic disease 2
MFI: Mean fluorescent intensity
PrP^C^: Cellular prion protein
SSC: Side-scatter (cell complexity measurement)
