# Supplementary material for: Histamine stimulates human microglia to alter cellular prion protein expression via the HRH2 histamine receptor
Source: Sci Rep. 2024 Oct 26;14:25519. doi: 10.1038/s41598-024-75982-1 (PMC11513956; doi:10.1038/s41598-024-75982-1)
Supplement: Supplementary file 1 — Supplementary Material 1 [file 41598_2024_75982_MOESM1_ESM.docx]

**Supplementary Figures**


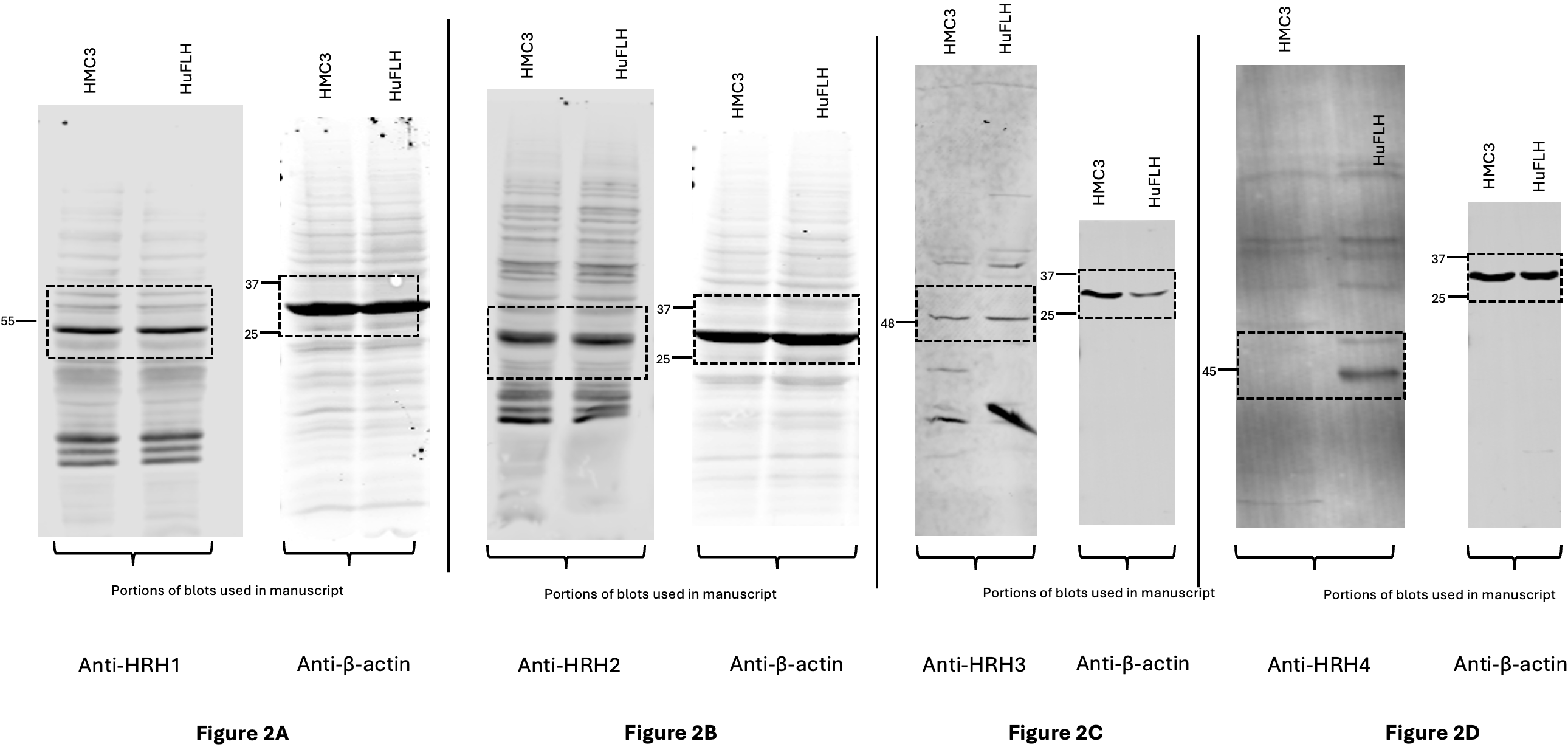


**Supplementary A.** Uncropped western blot images for Figure 2A-D. Gel electrophoresis and transfer were performed before slicing membranes and western blotting with anti-histamine receptor antibodies.


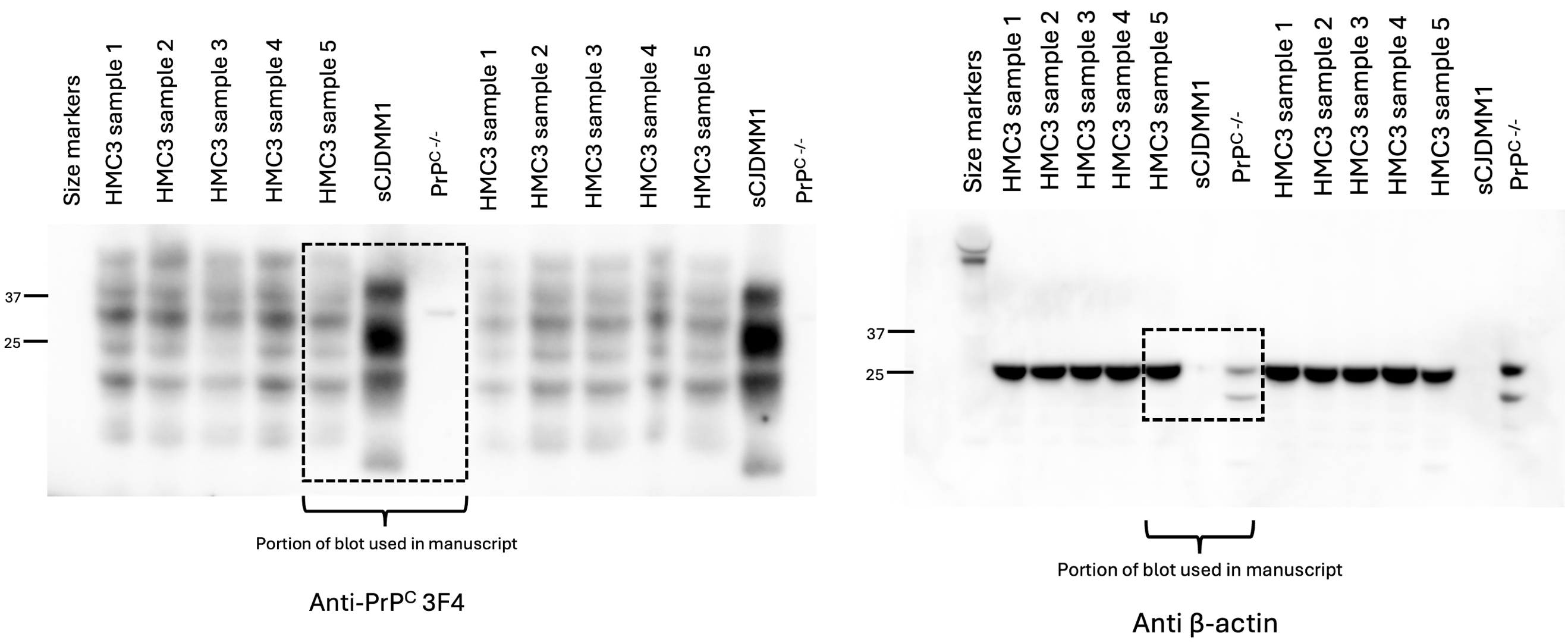


**Supplementary B.** Uncropped western blot images for Figure 4C.


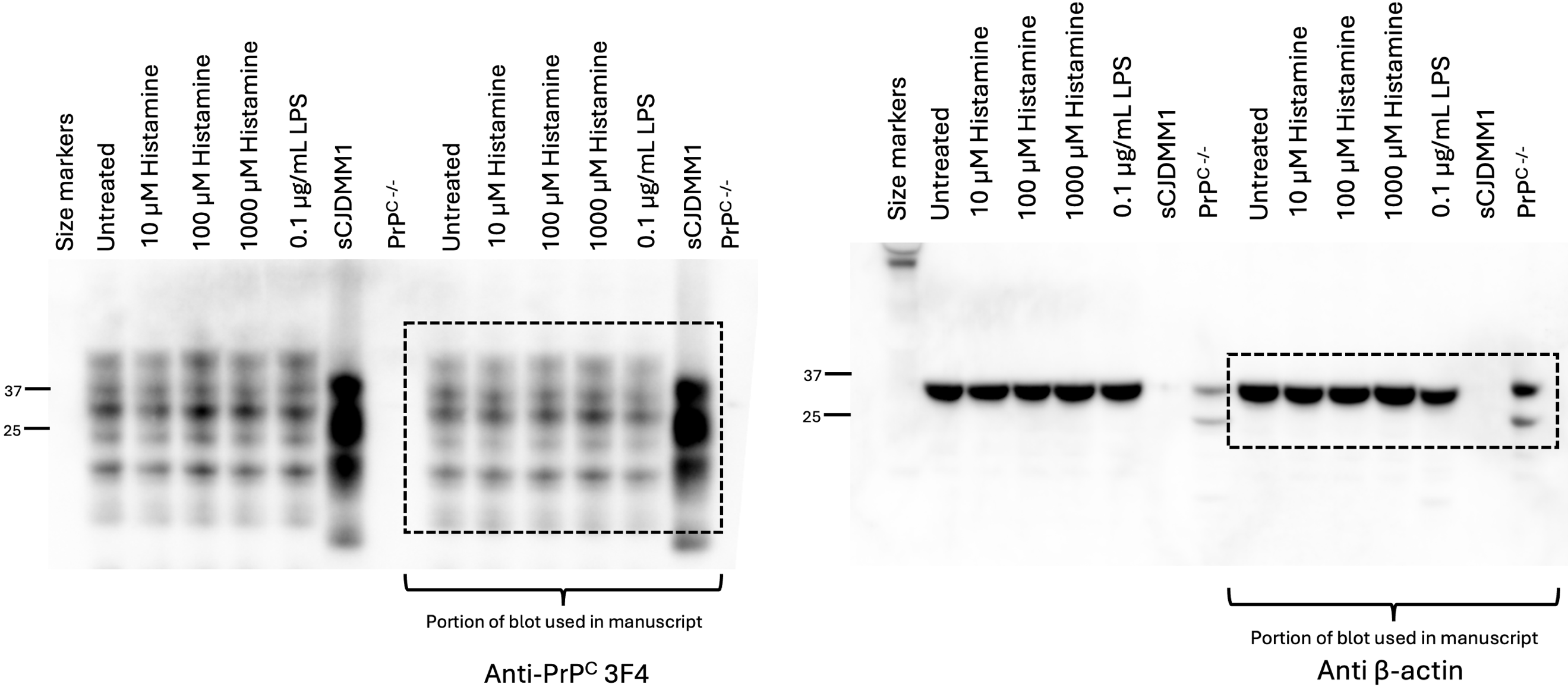


**Supplementary C.** Uncropped western blot images for Figure 6A.
