# Supplementary material for: Histamine stimulates human microglia to alter cellular prion protein expression via the HRH2 histamine receptor
Source: Sci Rep. 2024 Oct 26;14:25519. doi: 10.1038/s41598-024-75982-1 (PMC11513956; doi:10.1038/s41598-024-75982-1)
Supplement: Supplementary file 2 — Supplementary Material 2 [file 41598_2024_75982_MOESM2_ESM.docx]

**Histamine stimulates human microglia to alter cellular prion protein expression via the HRH2 histamine receptor**

Marcus Pehar^1,2,3^, Melissa Hewitt^4^, Ashley Wagner^1^, Jagdeep K. Sandhu^4^, Aria Khalili^1,5^, Xinyu Wang^1,5^, Jae-Young Cho^1,5^, Valerie L. Sim^2,6^, and Marianna Kulka^1,7^

^1^ Quantum and Nanotechnologies Research Centre, National Research Council Canada, Edmonton, Alberta, Canada

^2^ Neuroscience and Mental Health Institute, Faculty of Medicine and Dentistry, University of Alberta, Edmonton, Alberta, Canada

^3^ Centre for Prions and Protein Folding Diseases, University of Alberta, Edmonton, Alberta, Canada

^4^ Human Health Therapeutics Research Centre, National Research Council Canada, Ottawa, Ontario, Canada

^5^ Department of Mechanical Engineering, University of Alberta, Edmonton, Alberta, Canada

^6^ Department of Medicine, University of Alberta, Edmonton, Alberta, Canada

^7^ Department of Medical Microbiology and Immunology, University of Alberta, Edmonton, Alberta, Canada

**SUPPLEMENTARY FIGURES**


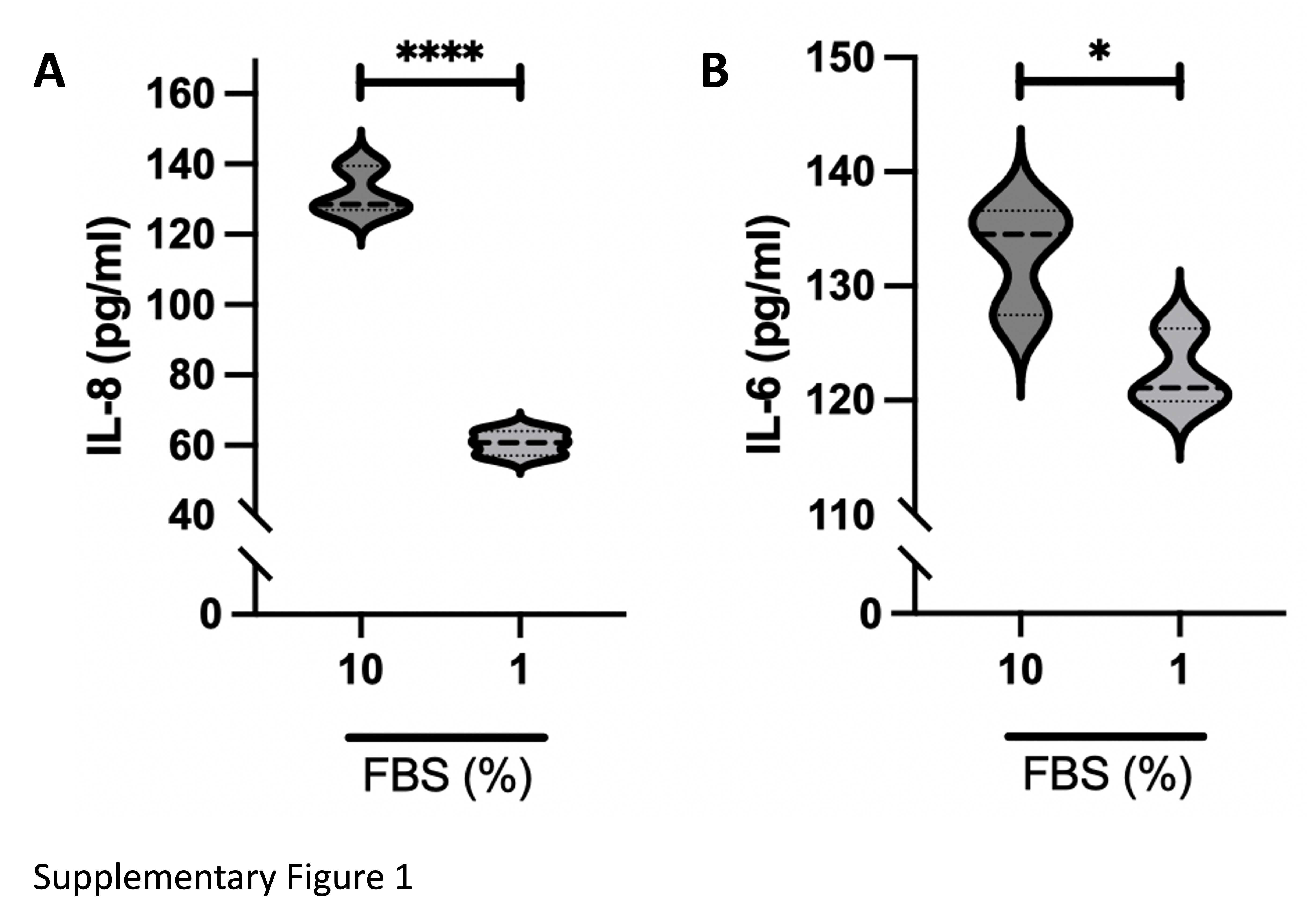


**Supplementary 1. HMC3 cells grown in media with lower serum concentrations release less IL-8 and IL-6.** HMC3 were cultured in complete media consisting of MEM, 10% FBS, 100 U/mL penicillin, and 100 µg/mL streptomycin. Twenty-four hours prior to supernatant collection, complete MEM media was replaced with media containing 1% FBS or complete media. (**A**) IL-8 and (**B**) IL-6 release were measured by sandwich ELISA. Data are presented as the mean ± SEM and statistical significance was measured via Student’s t-test, *p* ≤ 0.05 (*), *p* ≤ 0.0001 (****). (N=3).

**
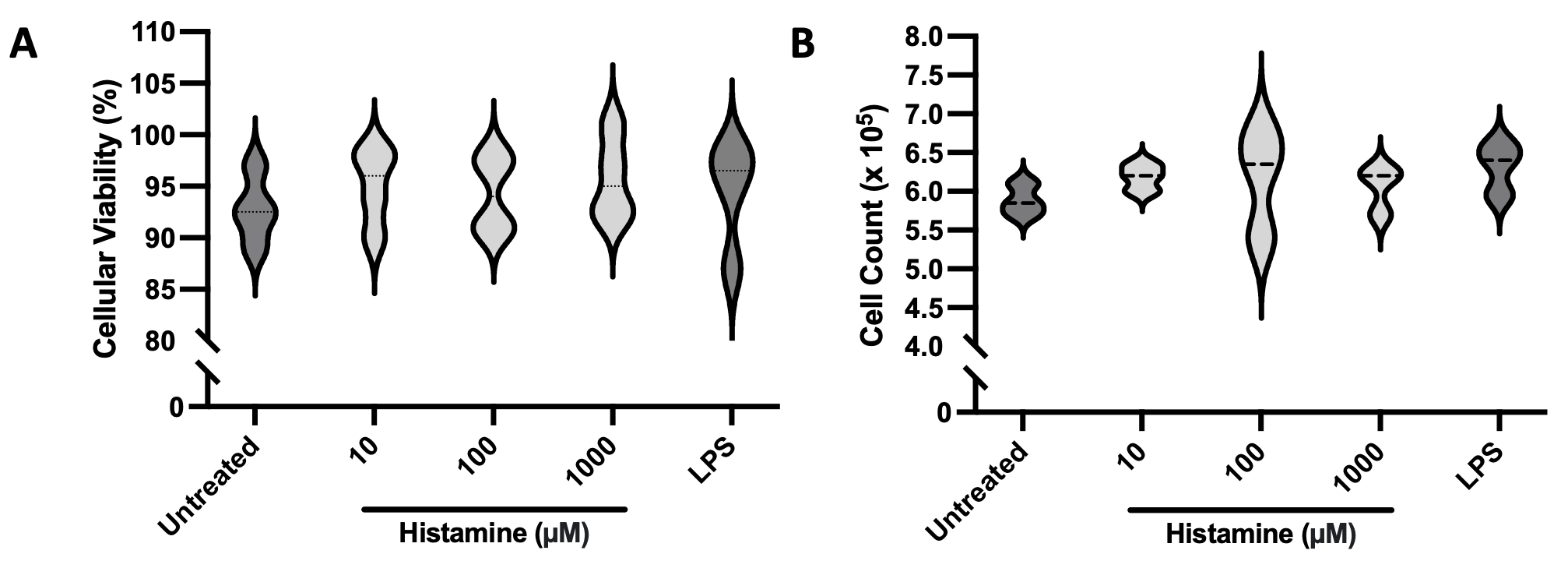
**

**Supplementary 2. HMC3 cells do not alter cellular viability or cell proliferation following histamine or LPS stimulation.** Cells were treated with histamine (10, 100, or 1000 μM) or LPS (1 μg/mL) for 24 hours and (**A**) cellular viability and (**B**) cell proliferation were assessed manually. Counts were performed via a single-blinded method. (N=4).


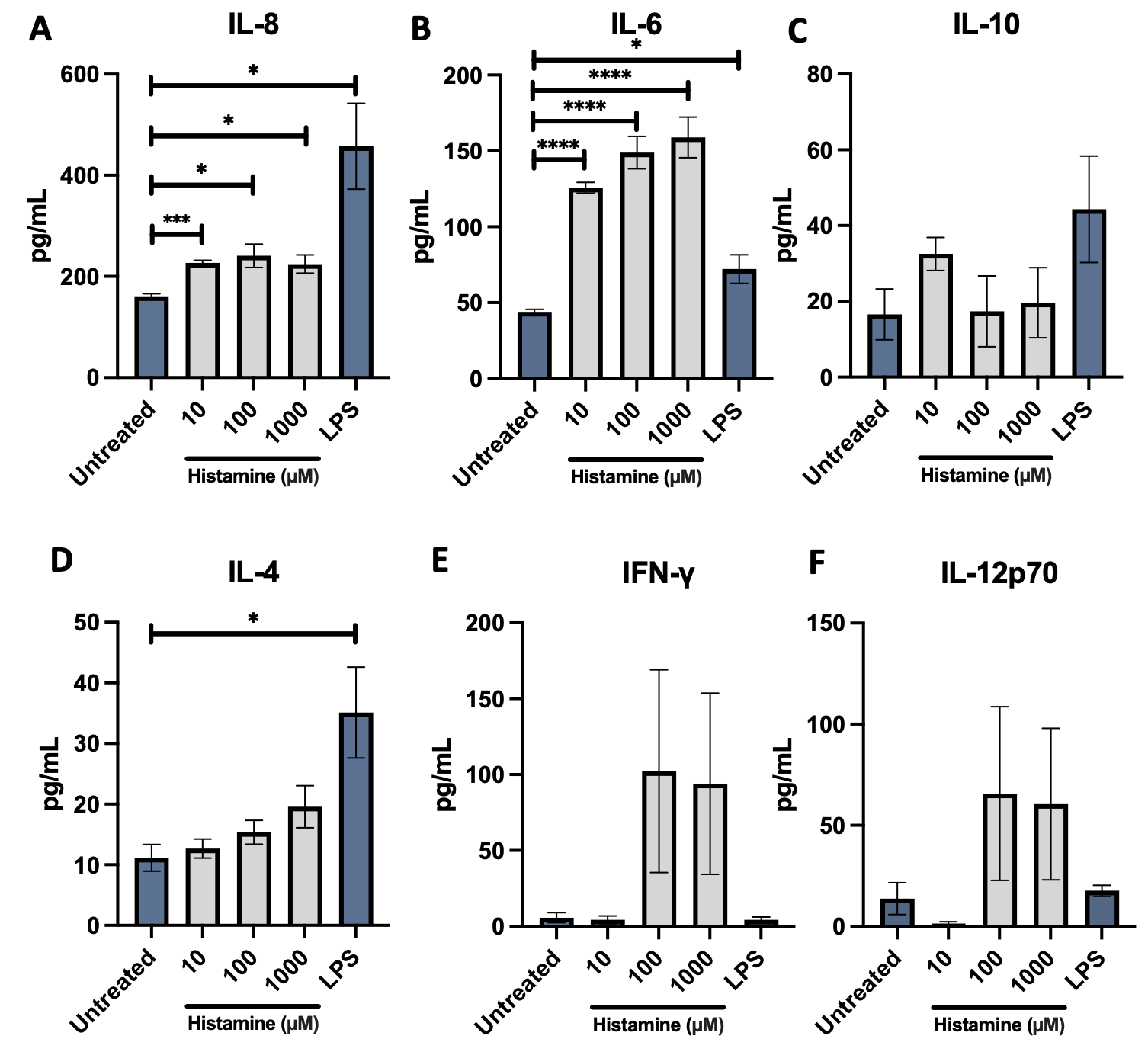


**Supplementary 3. HMC3 cells alter cytokine production following stimulation by histamine and LPS.** An electrochemiluminescence assay performed to measure cytokine release following histamine-induced stimulation showed that HMC3 altered the production of (**A**) IL-8, (**B**) IL-6, (**C**) IL-10, (**D**) IL-4, (**E**) IFN-γ, and (**F**) IL-12p70. (N=2, bars indicate ± SEM).

**
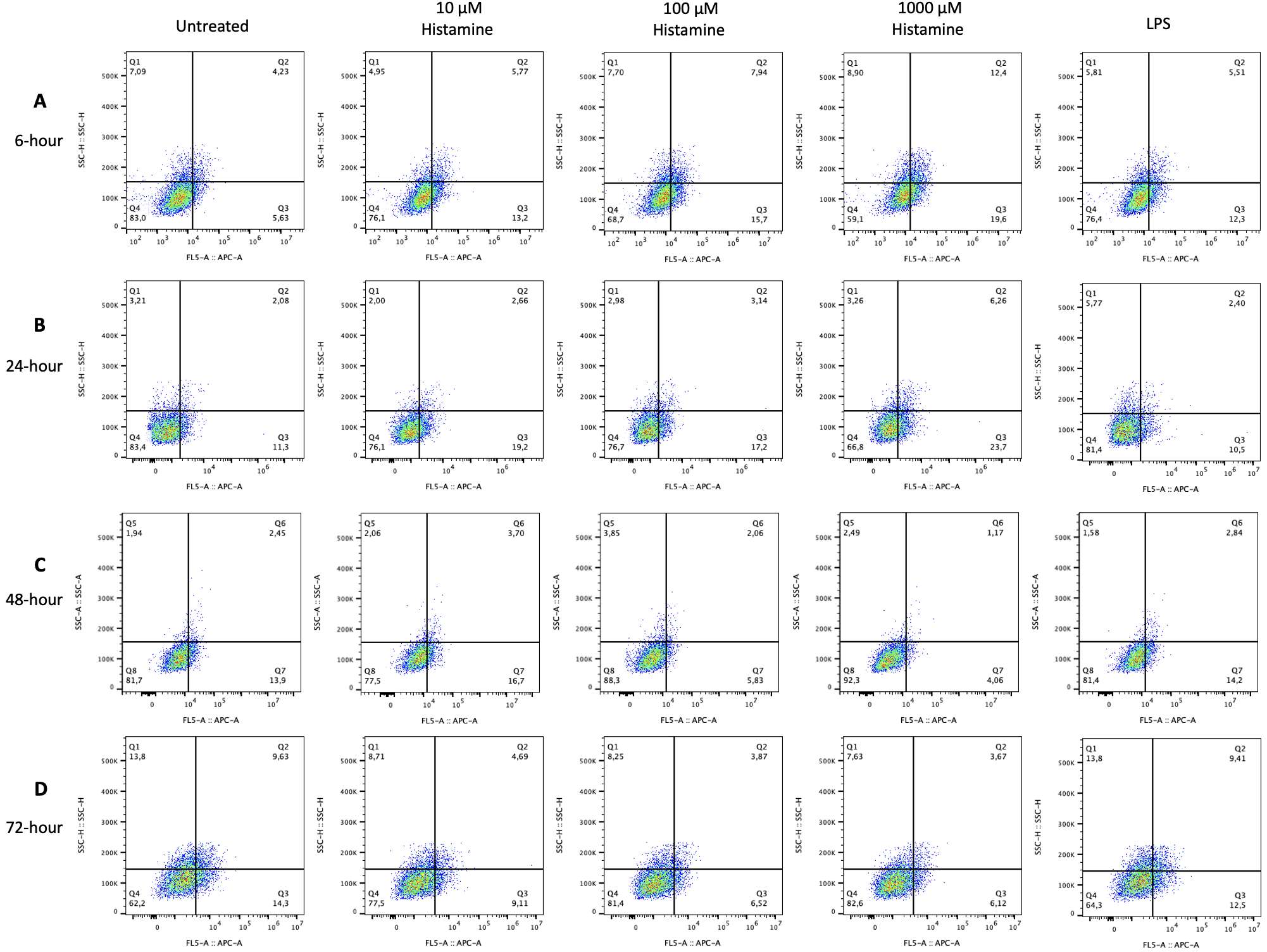
**

**Supplementary 4**. **Scatterplot representation of HMC3 cell complexity relative to PrP^C^ following histamine treatment for various periods of time.** Flow cytometry of HMC3 following treatment with 10, 100, or 1000 μM of histamine, 1 μg/mL of LPS, or no treatment. HMC3 were treated with histamine or LPS for (**A**) 6 hours, (**B**) 24 hours, (**C**) 48 hours, or (**D**) 72 hours. Graphs plot side scatter (y-axis) against PrP^C^ expression (x-axis). The percentages in each corner reflect the population of cells in each quadrant. Side-scatter (SSC) indicates cell complexity. (N=4).

**
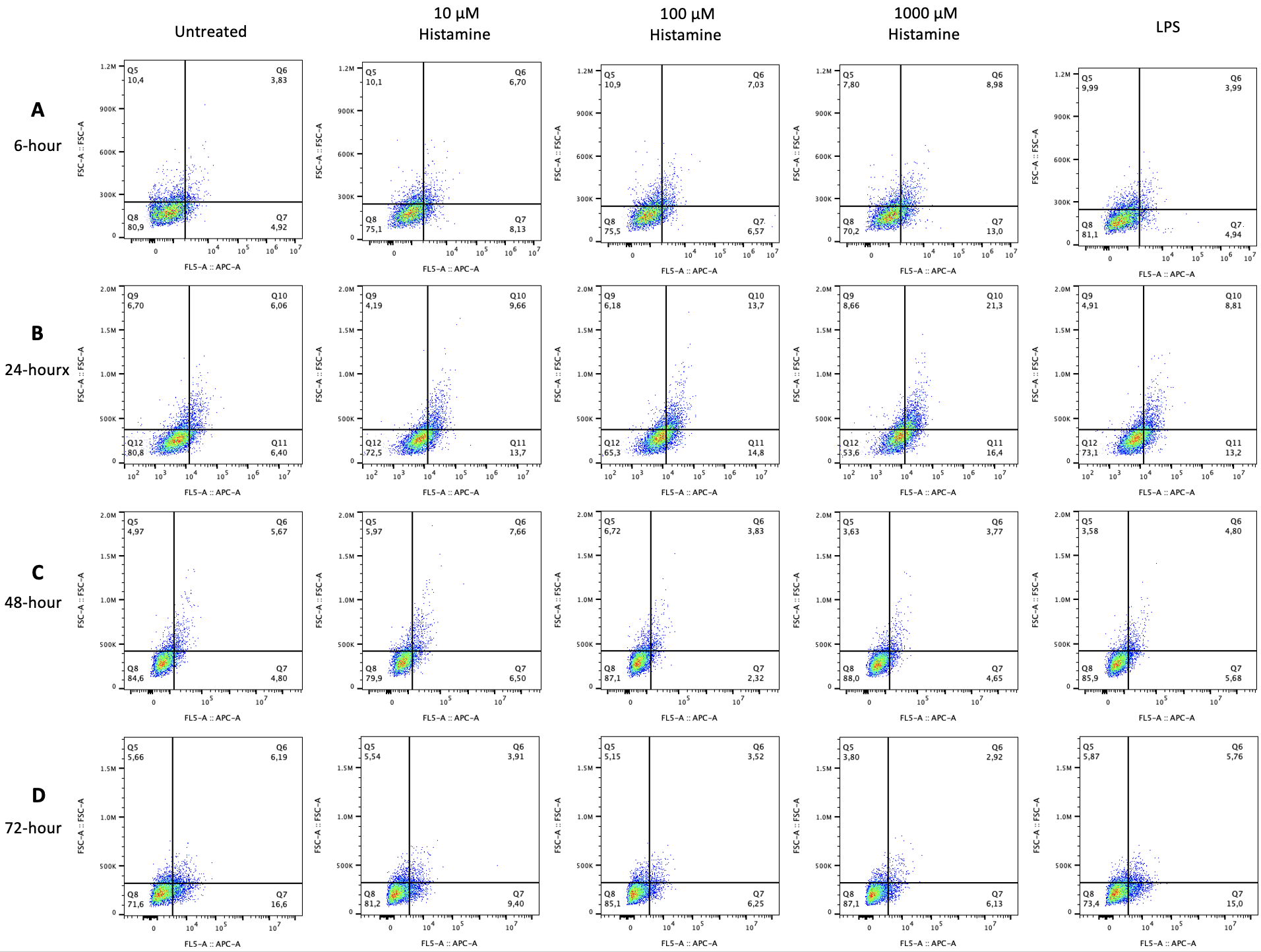
**

**Supplementary 5.** **Scatterplot representation of HMC3 cell size relative to PrP^C^ following histamine treatment for various periods of time.** Flow cytometry of HMC3 following treatment with 10, 100, or 1000 μM of histamine, 1 μg/mL of LPS, or no treatment. HMC3 were treated with histamine or LPS for (**A**) 6 hours, (**B**) 24 hours, (**C**) 48 hours, or (**D**) 72 hours. Graphs plot forward scatter (y-axis) against PrP^C^ expression (x-axis). The percentages in each corner reflect the population of cells in each quadrant. Forward-scatter (FSC) indicates cell size. (N=4).

**
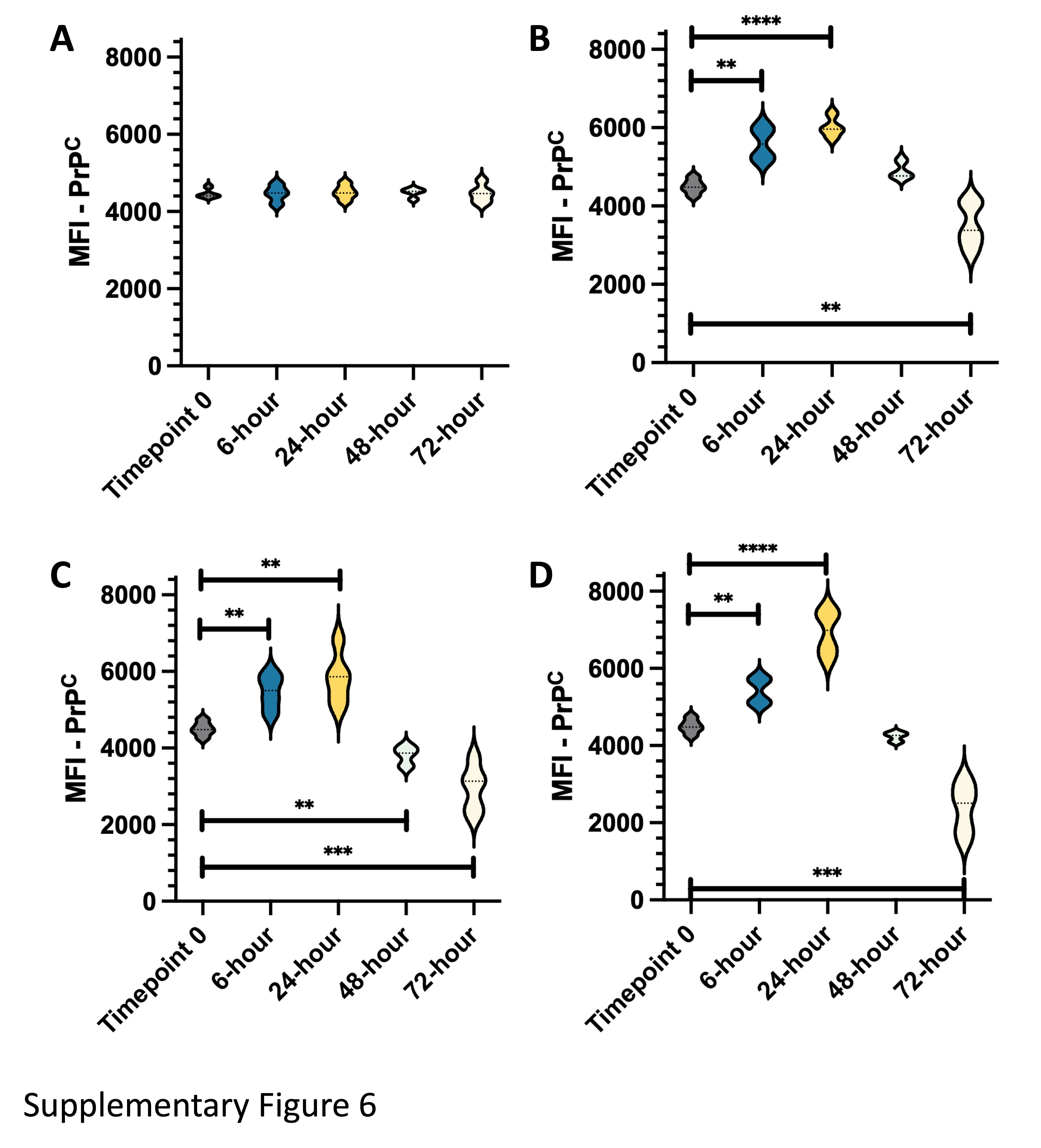
**

**Supplementary 6. HMC3 cells altered PrP^C^ expression following histamine-induced stimulation.** (**A**) Untreated HMC3 cells do not alter cell surface PrP^C^ over time. HMC3 decrease cell surface PrP^C^ following in a time-dependent manner following (**B**) 10 μM, (**C**) 100 μM, and (**D**) 1000 μM histamine treatment. Statistical significance was calculated using Student’s t-test, *p* ≤ 0.05 (*), *p* ≤ 0.01 (**), *p* ≤ 0.0001 (****). (N=4).


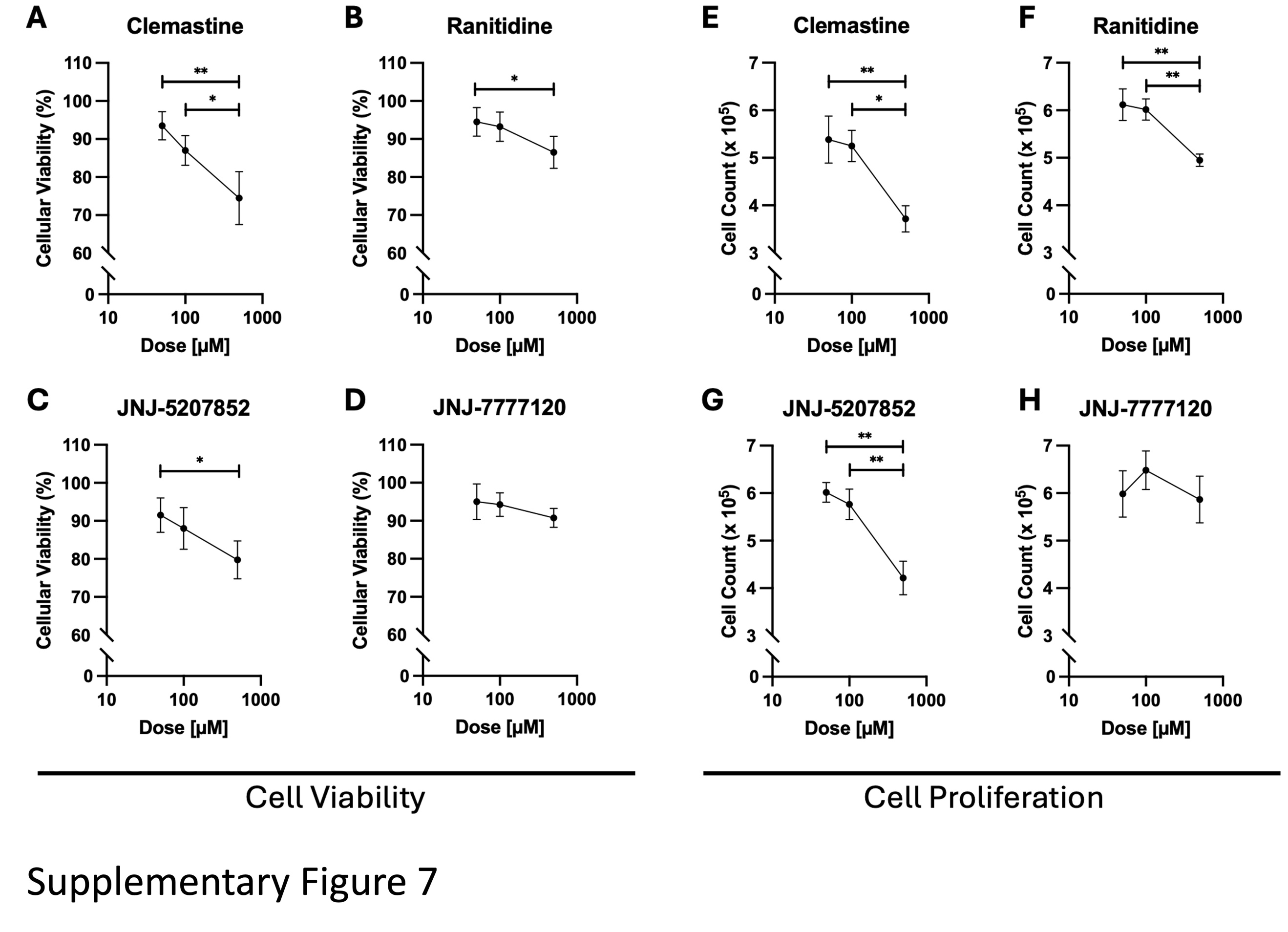


**Supplementary 7. HMC3 cells alter cellular viability and proliferation following treatment with high doses of histamine receptor antagonists.** Cells were treated with 50, 100, or 500 μM of clemastine, ranitidine, JNJ-5207852, or JNJ-7777120 for 24 hours and (**A**) cellular viability and (**B**) cell proliferation were assessed manually. Counts were performed via a single-blinded method. Data are presented as a dose-response curve with the mean ± SEM and statistical significance was measured via Student’s t-test, *p* ≤ 0.05 (*), *p* ≤ 0.01 (**). (N=4).

**
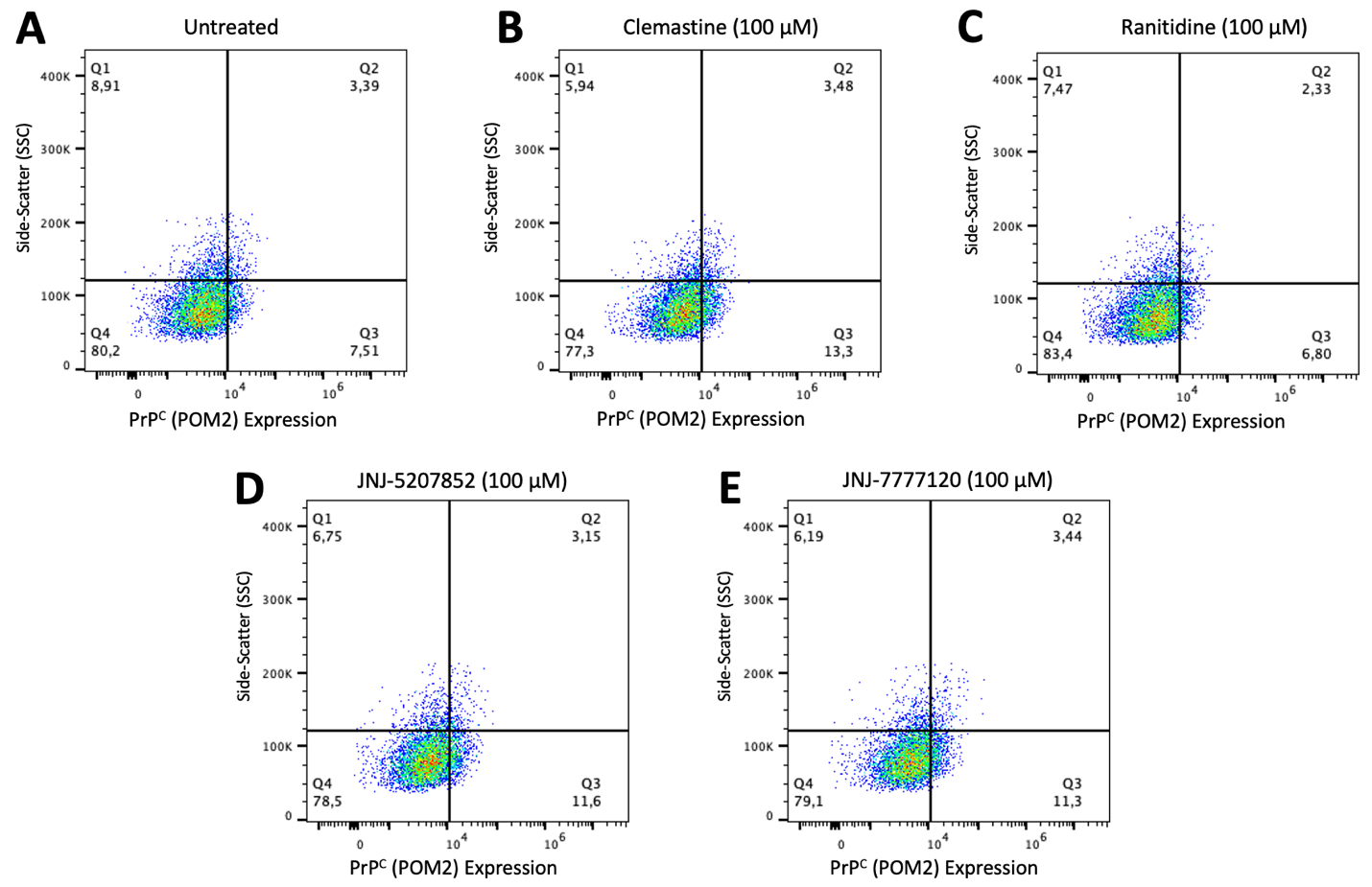
**

**Supplementary 8. Scatter-plot representation of HMC3 cell complexity relative to PrP^C^.** HMC3 cells were treated with individual histamine receptor antagonists for 1 hour followed by 100 μM histamine for 24 hours. Cell complexity (y-axis) was plotted relative to PrP^C^ expression (x-axis). (**A**) Untreated HMC3. (**B**) HRH1 inhibition by Clemastine. (**C**) HRH2 inhibition by Ranitidine. (**D**) HRH3 inhibition by JNJ-5297852. (**E**) HRH4 inhibition by JNJ-7777120. Side-scatter (SSC) indicates cell complexity. (N=4).


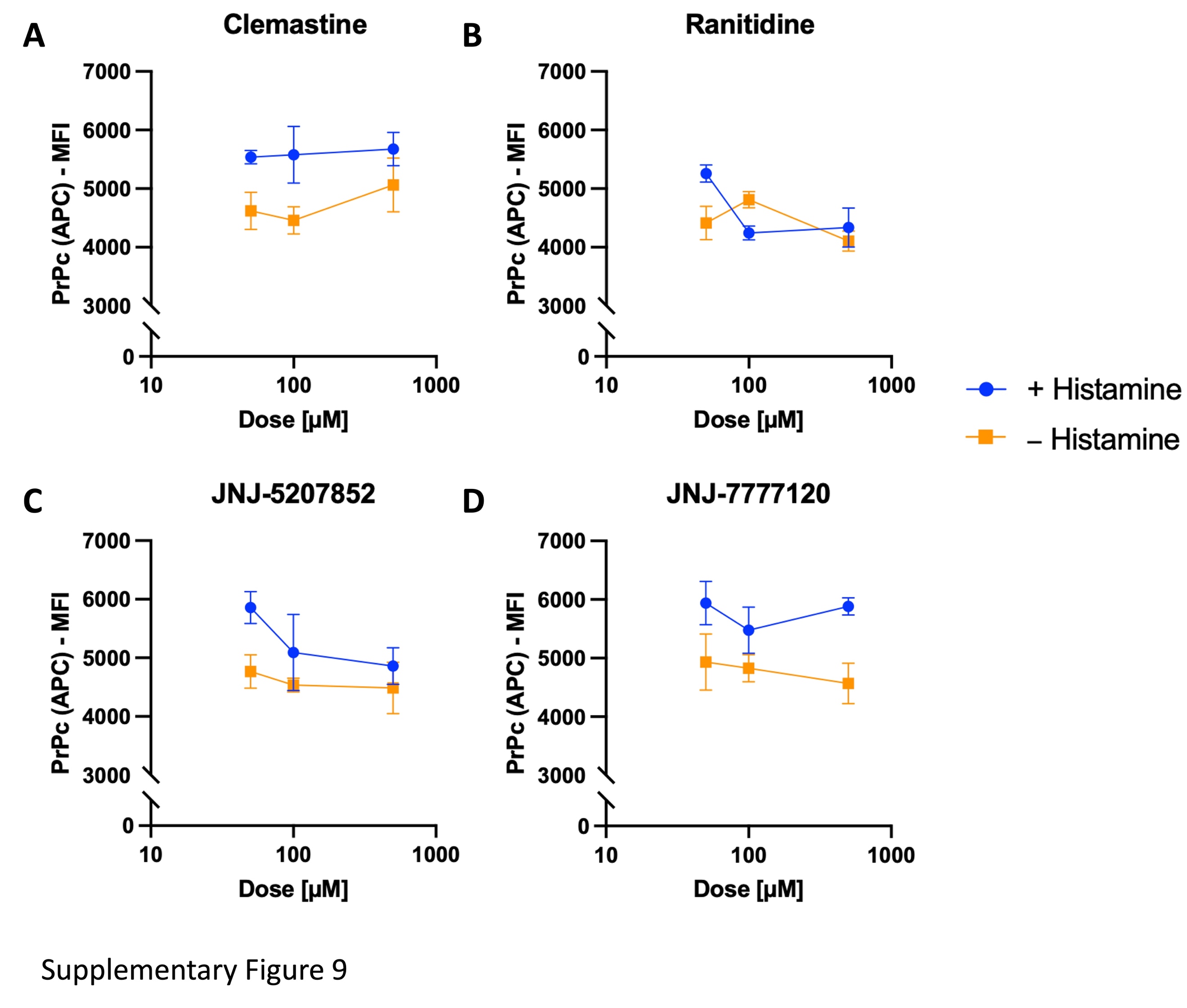


**Supplementary 9. Dose response curves of histamine receptor antagonists.** HMC3 cells were treated with 50, 100, or 500 μM (**A**) clemastine, (**B**) ranitidine, (**C**) JNJ-5207852, or (**D**) JNJ-7777120 for 1 hour and then either treated with 100 μM histamine (blue) or left untreated (orange) for 24 hours. Subsequently, PrP^C^ expression was measured by flow cytometry. Graphs are plotted as a dose response curve. (N=3).


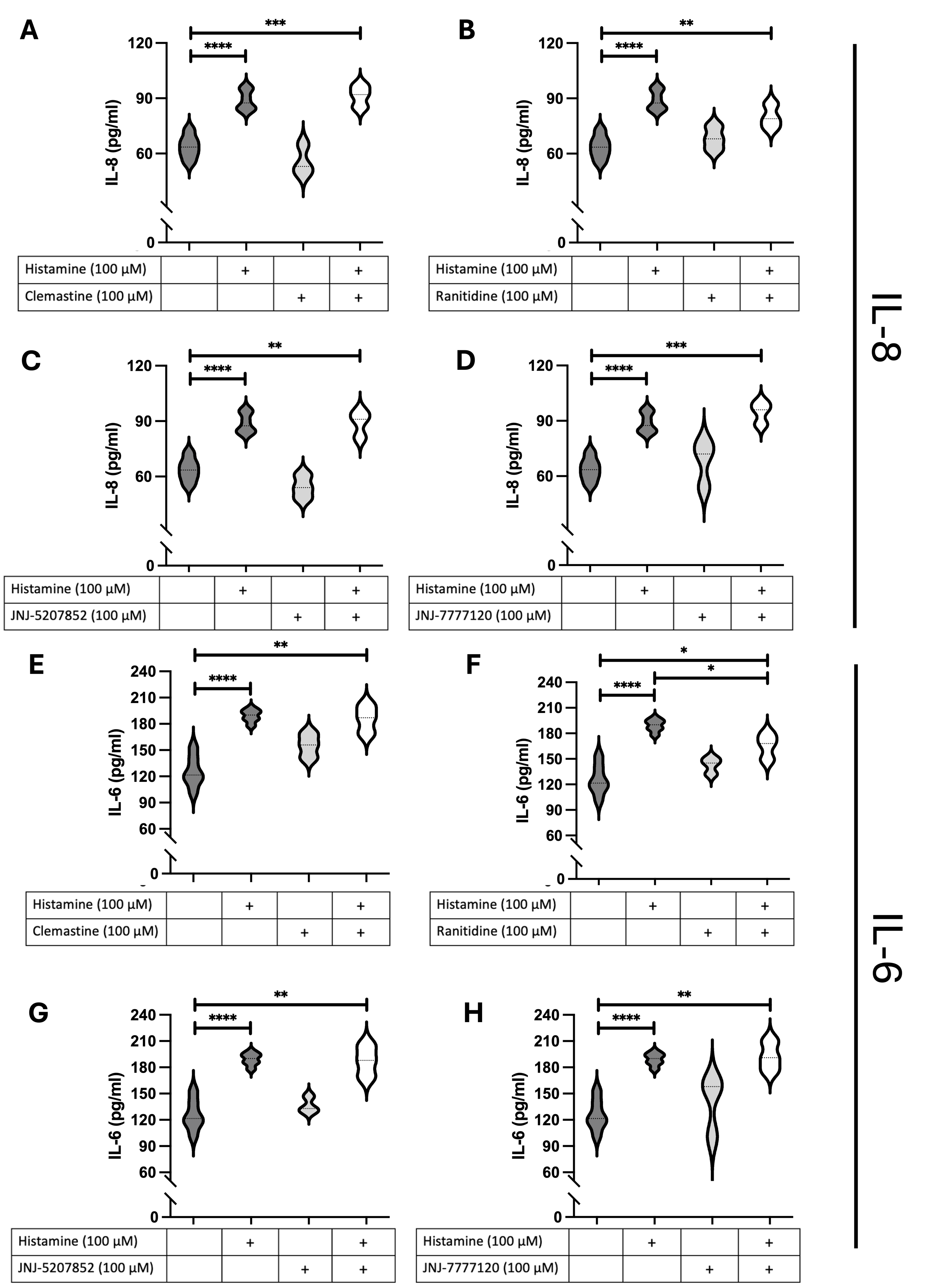


**Supplementary 10. Cytokine release of HMC3 cells following histamine receptor inhibition.** HMC3 cells were treated with individual 50, 100, or 500 μM of histamine receptor antagonists for 1 hour and then either treated with 100 μM histamine or treated with vehicle (PBS) for 24 hours. IL-8 and **(D)** IL-6 were measured by sandwich ELISA. Data are presented as the mean ± SEM (N=3) and statistical significance was measured via one-way ANOVA and Dunnett’s multiple comparison post-hoc analysis relative to untreated (UT) cells. *p* ≤ 0.05 (*), *p* ≤ 0.01 (**), *p* ≤ 0.001 (***), *p* ≤ 0.0001 (****).
